# MDA-MB-231 breast cancer cells adapted to anchorage-independent growth reveal senescent-like phenotype and persistent downregulation of PD-L1 expression

**DOI:** 10.3389/fonc.2025.1667308

**Published:** 2025-10-01

**Authors:** Tadeja Snedec, Jernej Repas, Darin Lah, Špela Zemljič, Mojca Pavlin

**Affiliations:** Institute of Biophysics, Faculty of Medicine, University of Ljubljana, Ljubljana, Slovenia

**Keywords:** triple-negative breast cancer, 2-deoxy-D-glucose, anchorage-independent growth, metastasis, PD-L1, senescence, epithelial-to-mesenchymal transition, mitochondria

## Abstract

**Introduction:**

Metastasis remains the primary cause of mortality in breast cancer, particularly in aggressive triple-negative (TNBC) subtypes. A crucial yet understudied aspect of this process involves cancer cell survival in a detached state and subsequent reattachment at distant sites. While *in vitro* models can capture early detachment events like tumor budding, the subsequent reattachment phase is often overlooked. To address this, we developed a TNBC cell model that effectively mimics metastatic behavior following re-attachment at secondary locations.

**Methods:**

We generated clones of MDA-MB-231 TNBC cells through consecutive selection for enhanced anchorage-independent survival. This was achieved using 2-deoxy-D-glucose (2DG) alone, 2DG combined with metformin, or Poly(2-hydroxyethyl methacrylate) (polyHEMA)-coated surfaces. The resulting adapted clones were characterized by RNA transcriptomics, comparing them to one-time treated non-selected cells. Real-time cellular energy metabolism was assessed via Seahorse analysis, while mitochondrial mass and intracellular localization were determined by flow cytometry and fluorescent microscopy, respectively.

**Results:**

Transcriptomic analysis of the MDA-MB-231 clones adapted to anchorage-independent growth revealed distinct alterations in gene expression, confirmed by principal component analysis showing clear separation from control clusters. These adapted clones exhibited increased mesenchymal and stemness markers, downregulated cell cycle genes, and a senescent-like phenotype. Interestingly, while nuclear-encoded oxidative phosphorylation (OxPhos) genes were downregulated, mitochondrial-encoded OxPhos genes were upregulated, with no significant shift in overall ATP production as measured by Seahorse analysis. Paradoxically, despite increased *CD274* (PD-L1) transcription, surface PD-L1 expression was consistently reduced, likely due to endoplasmic reticulum (ER) stress and impaired N-glycosylation.

**Discussion:**

Our adapted clones provide a novel *in vitro* model of early metastatic behavior, unveiling a cancer cell survival strategy that balances energy metabolism with adaptation to stress. These clones also demonstrated enhanced detachment properties and upregulation of proto-oncogenes, in addition to their senescent-like phenotype. Critically, the uncoupling of *CD274* transcription from surface PD-L1 expression suggests a potential therapeutic vulnerability that could be exploited in TNBC.

## 1 Introduction

Breast cancer is the most prevalent cancer among women worldwide and the second leading cause of cancer-related deaths. Tumor metastasis remains the primary cause of morbidity and mortality in breast cancer (1), with triple negative breast cancer (TNBC) representing one of the most aggressive subtypes and being associated with a poor prognosis (2). The leading hypothesis describing formation of metastasis is epithelial to mesenchymal transition (EMT), which proposes that cancer cells adopt a mesenchymal phenotype during an intermediate step (3). This phenotype is characterized by the loss of cell polarity, suppression of epithelial gene expression, and enhanced motility and invasiveness (4–6). However, because this process is difficult to observe *in vivo*, dynamics of EMT and its reversal to epithelial phenotype upon re-attachment (MET) remains incompletely understood.

A crucial step in metastasis formation on a cellular level is cell detachment from primary tumor and survival in an anchorage-independent state (3, 7) where cells must evade anoikis (8) - a form of cell death, triggered when epithelial cells lose contact with the extracellular matrix (ECM) (9, 10). Acquiring anoikis resistance is essential for metastasis and consists of multiple mechanisms, including integrin and metabolic alterations and activation of pro-survival, proliferative signaling (9, 11–18). One of crucial pathways that support anoikis resistance is the elevation of reactive oxygen species (ROS), which can activate the SRC pathway (19) and inhibit anoikis through ERK signaling (20, 21). In triple negative breast cancer cell line, such as MDA-MB-231, as well as in other cancers, upregulation of fatty acid oxidation (FAO) has been shown to be crucial for survival during anchorage-independent growth both *in vitro* and *in vivo* (22–31).

The *in vivo* analysis of cancer cell detachment during the metastatic cascade remains a significant challenge. While this process can sometimes be observed as tumor budding, circulating tumor cells (CTCs) represent an intermediate stage of metastasis. CTCs are characterized by low proliferation rate, high expression of stemness and mesenchymal markers and therefore resistance to conventional anti-cancer drugs (32–39). CTCs are a well-established prognostic factor for patient survival in breast cancer and other malignancies (40–43). At the time of metastasis diagnosis, these CTCs have already entered the bloodstream and are potentially colonizing new locations. This highlights a crucial therapeutic window: exploring strategies to inhibit cell re-attachment and colonization could be an effective way to prevent further metastatic spread (44).

Some studies (16, 45–49) including ours (50–52), have demonstrated that detachment can be induced *in vitro* through specific treatments or by forcing the cells to grow in anchorage-independent state using specially coated plates, such as poly(2-hydroxyethyl methacrylate) (polyHEMA). We have shown that viable cancer cells from various breast cancer cell lines and a colon cancer cell line can undergo detachment and anoikis resistance when treated with N-glycosylation inhibitors like 2-deoxy-D-glucose (2DG) and tunicamycin alone or in combination with metformin (Met) (50, 52, 53), while retaining their proliferative capacity. 2DG, a competitive inhibitor of glycolysis and N-glycosylation inhibitor, is being investigated as a potential anti-cancer agent (54–62), whereas Met, a widely used antidiabetic drug, has also demonstrated anticancer properties (63–68).

While some early steps of detachment can be observed as tumor budding in histopathological examinations (35, 36), the mechanisms that enable survival in an anchorage-independent state and subsequent reattachment, remain poorly understood due to the challenges of studying this process *in vivo*. In addition to the limited understanding of cancer metastasis, a major obstacle to successful treatment is resistance of metastatic and CTC cells to conventional anti-cancer therapies. Therefore, gaining deeper insight into metastasis formation and the surviving mechanisms of cells under anchorage-independent conditions is crucial for improving cancer treatments and reducing mortality.

In this study, we generated a clone of MDA-MB-231 triple negative breast cancer cells selected for their ability to survive under anchorage-independent conditions. The cells were obtained by selecting the detached population following the specific treatments (2DG alone or in combination with Met) or by culturing them on polyHEMA-coated surface. To establish a subpopulation of detached cells, we repeatedly reseeded only the detached cells over multiple cycles, ultimately obtaining a cell subtype better adapted to anchorage-independent survival - partially mimicking cancer metastasis. We then analyzed both the parental wild-type cells and the adapted clones to identify the mechanisms that enable detached cells to survive in the anchorage-independent state, reattach, and proliferate in the compound free medium. Additionally, transcriptomic analysis was performed to investigate differences in metabolism, signaling pathways and cell cycle regulation. Our *in vitro* model can be used to conceptually parallel the metastatic cascade by recreating its key stages. The initial detachment of cells corresponds to the process of cancer cell detachment. The detached cells represent circulating tumor cells (CTCs), while subsequent re-attachment reflects the colonization at a secondary site.

## 2 Materials and methods

### 2.1 Antibodies and reagents

Metformin was obtained from Calbiochem (Merck Millipore) and 2-deoxy-D-glucose from Santa Cruz Biotechnology (sc-3506). CM-H2DCFDA was obtained from Invitrogen. CellTak was obtained from Corning. Seahorse XF Real-Time ATP Rate Assay Kit was obtained from Agilent. RPMI 1640 medium was obtained from Genaxxon Bioscience. All other reagents, unless otherwise specified, were purchased from Sigma-Aldrich or Merck Millipore.

### 2.2 Cell culture and treatments

MDA-MB-231 cell line was purchased from ATCC (USA). Cells were routinely grown in RPMI-1640 medium (Genaxxon Bioscience) with 4.5 g/L (25 mM) glucose, 2 mM glutamine and 10% FBS in a humidified atmosphere with 5% CO_2_ at 37 °C. For all experiments, cells were seeded in complete RPMI-1640 with 4.5 g/L glucose for 24 h, washed with isotonic NaCl, and subsequently grown in RPMI-1640 medium supplemented with 10% FBS, 2 mM glutamine and 1 g/L (5.6 mM) glucose. All experiments were performed in a humidified atmosphere at 37 °C and 5% CO_2_. Nutrient availability largely dictates metabolic behavior (69), therefore, we used the physiological 1 g/L glucose, instead of hyperglycemic 4.5 g/L, that is usually used in cell culture.

### 2.3 Experimental design

MDA-MB-231 cells were plated on 6-well plates in RPMI-1640 medium supplemented with 4.5 g/L glucose, 2 mM glutamine and 10% FBS. On the next day, cells were washed with isotonic NaCl solution, and the medium was changed to complete RPMI medium with 1 g/L glucose. Cells were treated for 72 hours with 4.8 mM 2DG or co-treated with 5 mM Met and 0.6 mM 2DG. We have chosen 4.8 mM concentration of 2DG and combination of 5 mM Met + 0.6 mM 2DG for the selection process, based on our previous research, which showed significant detachment of viable MDA-MB-231 cells for these conditions, while at higher concentrations extensive cell death and reduced detachment were observed (50–52, 70). As an additional sample, cells were grown on polyHEMA-coated plates, which prevents cell attachment. Medium was renewed daily, and the detached cells were collected and returned to the attached cell counterpart. After 72 hours, attached and detached cells were collected and counted separately. Only the detached cells were reseeded in the complete RPMI medium with 4.5 g/L glucose. After 72 hours, cells were counted again and reseeded in complete RPMI-1640 medium with 4.5 g/L glucose, washed with isotonic NaCl the next day and treated with the same compounds as before for 72 hours. The whole cycle was repeated four times. After four cycles of detached cell selection, the detached and attached cells were collected separately, counted and the differences between treated adapted clones and wild-type cells were determined. The detached cells were also reseeded again in the complete RPMI medium with 4.5 g/L glucose. After 72 hours, cells were counted again and the differences between adapted clones and wild-type cells were determined.

### 2.4 Analysis of the percentage of total cell number and the percentage of detached cells

On the final day of the experiment, supernatants with detached cells were collected separately. Attached cells were trypsinized and harvested separately. Detached cells were centrifuged separately and resuspended in compound-free medium. Cells were stained by Trypan blue and detached and attached cells were counted separately using Countess cell counter (Invitrogen, USA). The total number of cells was calculated as a sum of attached and detached cells and was normalized to the number of seeded cells. The percentage of detached cells was calculated as the number of detached cells divided by the total number of cells.

### 2.5 Detection of reactive oxygen species using CM-H2DCFDA

On the final day of the experiment, supernatants with detached cells were collected separately. Attached cells were trypsinized and harvested separately. Both detached and attached cells were centrifuged 5 min at 290 rcf and resuspended in 4.17 µM CM-H2DCFDA in PBS (with Ca^2+^ and Mg^2+^). For positive control, 1.1 mM H_2_0_2_ was used. After 20 min incubation on 37 °C, cells were analyzed using Attune™ NxT flow cytometer (Thermo Fisher Scientific, Waltham, USA). About 4 × 10^4^ events per sample were collected by BL-1 filter (492/520). We used Attune Cytometric Software for final analysis.

### 2.6 Determination of mitochondrial mass

On the final day of the experiment, supernatants with detached cells were collected separately. Attached cells were trypsinized and harvested separately. Same amounts of cells were prepared per sample, centrifuged, resuspended in complete RPMI medium with 4.5 g/L glucose and with 200 nM Mitotracker Orange^®^ for 45 min at 37 °C. After staining, cells were centrifuged, washed and resuspended in PBS and analyzed using Attune™ NxT flow cytometer (Thermo Fisher Scientific). About 4 × 10^4^ events per sample were collected by BL-2 filter (554/76). We used Attune Cytometric Software for final analysis.

### 2.7 Detection of surface PD-L1

On the final day of the experiment, supernatants with detached cells were collected separately. Attached cells were detached using PBS + 2 mM EDTA. 50,000 of cells were prepared per sample, centrifuged and resuspended in 75 µL PBS + 5% FBS with 0.5 µL APC-conjugated anti-PD-L1 antibody (329707, Biolegend, San Diego, CA, USA). After 20 min incubation, cells were washed in PBS + 1% BSA and resuspended in ice-cold PBS. Cells were analyzed using Attune™ NxT flow cytometer. About 2 × 10^4^ events per sample were collected by RL-1A filter (670/14). We used Attune Cytometric Software for final analysis.

### 2.8 Real-time cell Mito stress test

MDA-MB-231 cells were plated on Seahorse XFe24 cell culture microplates at 20,000 cells per well in RPMI medium with 4.5 g/L glucose. After 24 h, cells were washed with isotonic NaCl solution and the medium was replaced with RPMI 1640 medium with 1 g/L glucose and treated with compounds as before, with a medium renewal after 24 h. After 48 h of treatment, the medium was replaced with the RPMI 1640-based Seahorse XF Glycolytic Rate Assay medium (2 mM glutamine, 1 mM HEPES, 0 mM pyruvate, 1 g/L glucose) equilibrated to pH 7.4, with the same concentrations of Met and 2DG as in the treatment media. For the detached cells, MDA-MB-231 (wild-type and clones) cells were plated in a cell culture flask and treated the same as cells on Seahorse XFe24 cell culture microplates. After 48 h of treatment, the detached cells were collected, centrifuged, and resuspended in RPMI 1640-based Seahorse XF Glycolytic Rate Assay medium (2 mM glutamine, 1 mM HEPES, 0 mM pyruvate, 1 g/L glucose) equilibrated to pH 7.4, with the same concentrations of Met and 2DG as in the treatment media, plated on Seahorse cell culture microplates covered with CellTak^®^ at 150,000 cells in 0.1 mL per well. Plates were spun down at 200 g for 1 min and incubated at 37 °C without CO_2_ for 15 min, after which 0.4 mL of Seahorse XF Glycolytic Rate Assay medium was added and after additional 30 min of incubation at 37 °C without CO_2_. OCR and ECAR were measured and the ATP production rate from glycolysis and OxPhos was determined according to Seahorse XF Cell Mito Stress Test protocol, by serial injections of following regents at final concentrations of 1 μM oligomycin, 8 μM carbonyl cyanide-p-trifluoromethoxyphenylhydrazone (FCCP) and 1 μM Rotenone/Antimycin A. Results were normalized to the total DNA content, determined by Hoechst staining.

### 2.9 Transcriptome sequencing

On the final day of the experiment, the reattached detached cells were trypsinized and harvested. Cells were washed twice with ice cold PBS, snap frozen in liquid nitrogen, and stored at – 80 °C. Total cell RNA was isolated from cell pellets using PureLink™ RNA Mini Kit (Invitrogen, ThermoFisher Scientific, Waltham, USA). Transcriptome sequencing was performed in triplicates in each group by Novogene (Cambridge, UK). Heatmaps were visualized using TBtools (71).

### 2.10 Confocal microscopy

Reattached clones CLONE^2DG^ and CLONE^polyHEMA^, as well as control cells were plated in chambered coverslips (IBIDI) in complete RPMI medium without added compounds. After 24 hours, cells were washed and labelled with Phalloidin Alexa Fluor 488 solution (Invitrogen) and 200 nM Mitotracker Orange^®^. After staining, cells were washed gently and the samples were examined under the Nikon ECLIPSE TE2000-E microscope (Plan Apo TIRF objective, magnification 60×, NA = 1.45) in the confocal mode (Nikon C1).

### 2.11 Statistical analysis

Results were displayed as mean ± SEM of three biological replicates unless indicated otherwise. Unless stated otherwise, one-way ANOVA with Dunnett’s *post hoc* test was used to test statistical significance of results. In some cases, two-way ANOVA was used to test for synergism between Met and 2DG treatment.

## 3 Results

**Figure 1:**
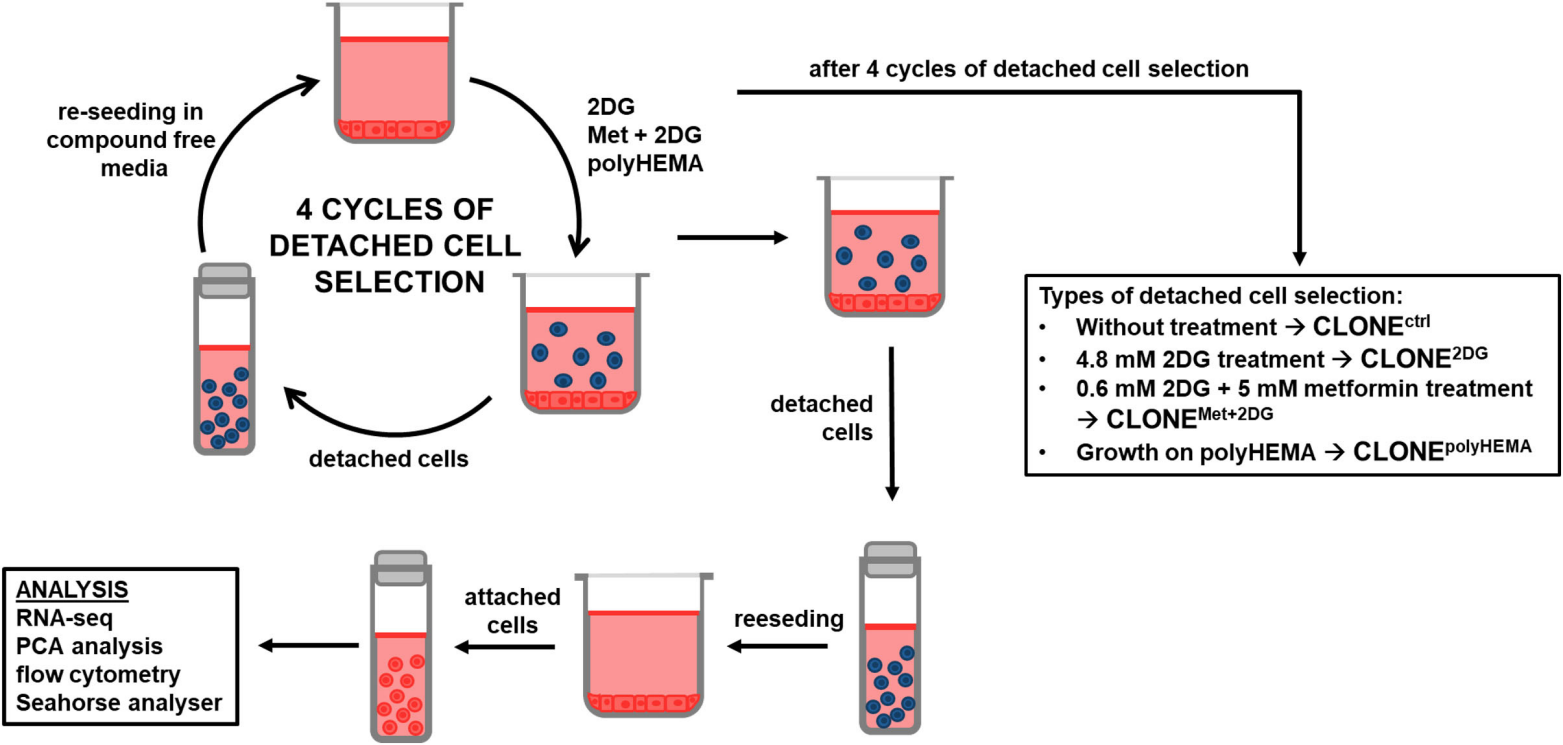
Schematic presentation of the experimental workflow. MDA-MB-231 cells were grown in RPMI 1640 media with 1 g/L glucose and treated with 2DG alone or in combination with metformin or grown on polyHEMA coated plates. After 72 hours, detached cells were reseeded in compound-free media for 72h. Reattached cells were reseeded again and treated with the same compounds. We have performed 4 cycles of detached cell selection. After the last cycle, attached and detached cells were collected and analyzed separately, the detached cells were again reseeded in compound free media and left to proliferate for 72 hours. Reattached cells were then analyzed. Met, metformin; 2DG, 2-deoxy-D-glucose.

**Figure 2:**
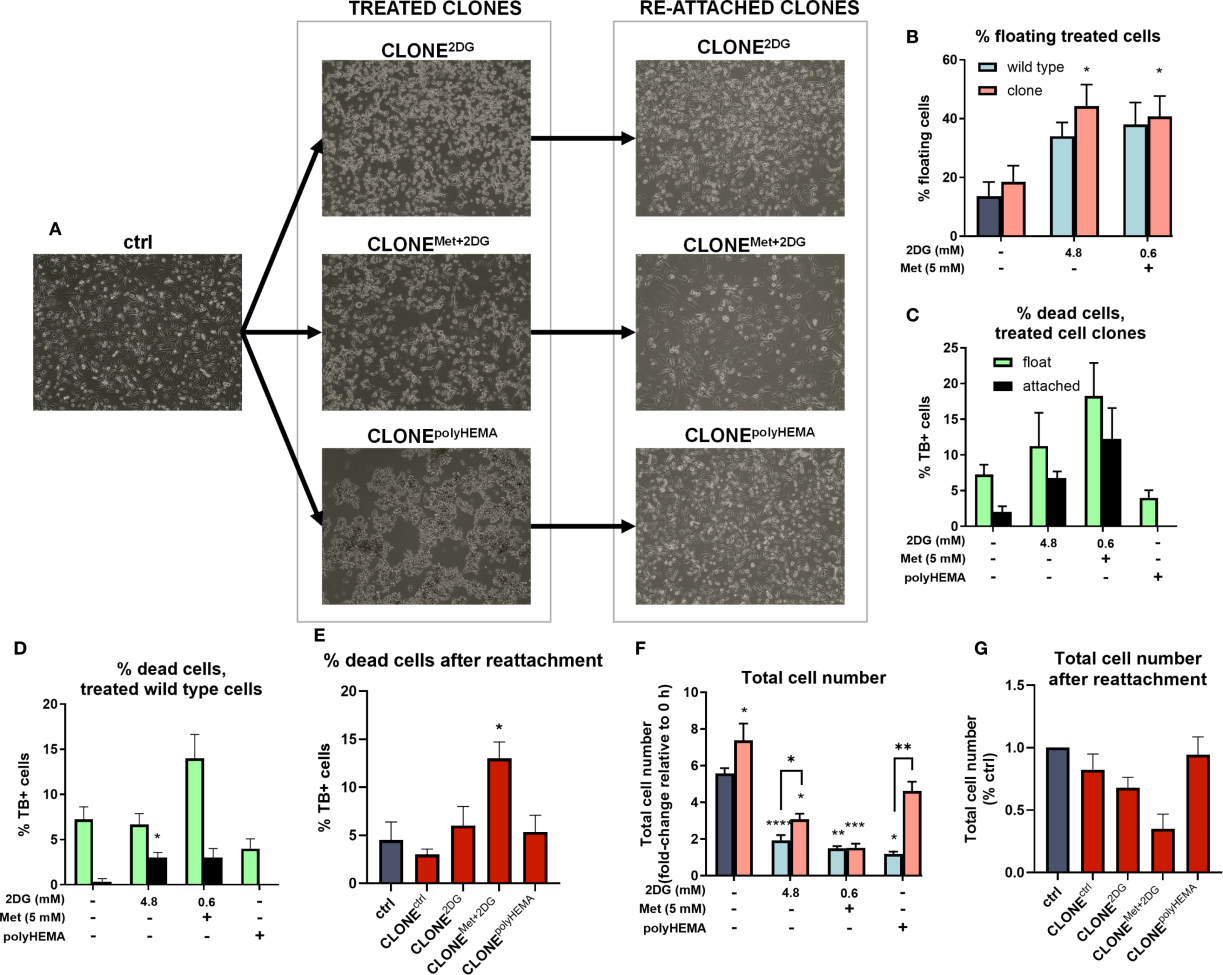
Percentage cell detachment and cell number of the adapted clones. MDA-MB-231 cells were grown in RPMI 1640 media with 1 g/L glucose and treated with 2-deoxy-D-glucose alone or in combination with metformin or grown on polyHEMA coated plates. After 72 hours, detached cells were reseeded in compound-free media for 72h. Reattached cells were reseeded again and treated with the same compounds. We have performed 4 cycles of detached cell selection. Representative cell images are shown in **(A)**. After the last cycle, detached and attached cells were stained with trypan blue counted separately with Countess cell counter and the percentage of floating cells **(B)**, % dead cells in treated adapted clones **(C)**, % dead cells in treated wild-type cells **(D)** and the total cell number **(F)** were determined. After the last cycle, the detached clones were reseeded again and left to re-attach. After 72 hours, cells were stained with trypan blue and counted with Countess cell counter and the % dead cells **(E)** and total cell number **(G)** after re-attachment were determined. The mean ± SEM for three independent experiments is shown. *p<0.05, **p<0.01, ***p<0.001, ****p<0.0001 versus (attached) control cells as determined by one-way ANOVA with Dunnett’s or Šidak’s *post-hoc* test. Met, metformin; TB, trypan blue; 2DG, 2-deoxy-D-glucose.

In this study we have generated a clone of MDA-MB-231 cells by repeatedly selecting the detached cell population following 4.8 mM 2DG (4.8 2DG) and 5 mM Met + 0.6 mM 2DG (Met+0.6 2DG) treatments. We have chosen the two specific treatments for the selection process, based on our extensive previous research, which showed significant detachment of MDA-MB-231 cells with preserved viability for these treatments. We observed a dose dependent hermetic effect with increasing percentage of viable floating cells up to a point when cell viability started to decrease (50–52, 70), with maximal detachment of viable cells for 4.8 2DG and Met+0.6 2DG treatments. As an additional condition, we cultured untreated MDA-MB-231 cells on polyHEMA-coated plates, which prevent cell attachment, to force the cells to grow under anchorage-independent state. The resulting adapted clones were named: CLONE^ctrl^, CLONE^2DG^, CLONE^Met+2DG^ and CLONE^polyHEMA^. In parallel, we prepared wild-type cells, by treating cells once and allowing them to reattach. The resulting cells were named WT^ctrl^ (untreated), WT^2DG^ (4.8 mM 2DG-treated), WT^Met+2DG^ (5 mM Met + 0.6 mM 2DG-treated) and WT^polyHEMA^ (grown on polyHEMA-coated plates). As a control group, untreated, attached MDA-MB-231 cells were re-seeded four times and referred to as ctrl. Experimental design is presented in **Figure 1**. Representative micrographs are shown in **Figure 2A**. Unless otherwise specified, all results represent comparison of clones compared to ctrl cells.

### 3.1 Selection of the detached population after 2DG treatment increases detachment

We hypothesized that repeated selection of the detached population of cells following treatment with 4.8 2DG and Met+0.6 2DG would enhance the detachment of viable cancer cells. We indeed observed an increasing trend in the percentage detached cells in CLONE^2DG^ compared WT^2DG^ cells treated with 4.8 2DG, however the increase was not statically significant (**Figure 2B**). Furthermore, treatment with 4.8 2DG led to an increase in cell death from 7% in WT^2DG^ to 11% in CLONE^2DG^ (**Figures 2C, D**). Similarly, treatment with Met+0.6 2DG increased cell death from 14% in WT^Met+2DG^ to 18% in CLONE^Met+2DG^ (**Figures 2C, D**). No significant differences in cell death were observed between wild-type and clonally selected cells grown on polyHEMA.

Importantly, we observed no significant reduction in cell viability within the floating cell populations of both adapted clones and wild-type cells (**Figures 2C, D**), indicating that most detached cells remained viable, with only a small proportion of dead detached cells. Moreover, analysis of the percentage of detached cells within the live cell population revealed that approximately 35% of cells were detached in CLONE^2DG^ and CLONE^Met+2DG^ (**Supplementary Figure S1**), further demonstrating high viability of detached cells in these adapted clones. Upon reattachment, cell death in CLONE^Met+2DG^ reached up to 12%, while in other clones, the proportion of dead cells remained around 5% (**Figure 2E**).

Next, we analyzed the impact of clonal adaptation on proliferation by analyzing the total cell number after 72h growth. Both CLONE^2DG^ and CLONE^Met+2DG^ showed a significant reduction in total cell number compared to ctrl (p<0.05) (**Figure 2F**).

Following we analyzed how the cells in CLONEs respond to metabolic inhibitors compared to WT cells. We compared the total cell number of treated clones to wild-type cells subjected to the same treatments (**Figure 2F**). For Met+0.6 2DG treatment, no significant differences in total cell number were observed between treated clones and wild-type cells. However, for 4.8 2DG treatment, CLONE^2DG^, and CLONE^polyHEMA^ cells (cells grown on polyHEMA-coated plates), exhibited a significant increase in total cell number compared to their respective wild-type counterparts (p<0.05 for both). Overall, in the Met+0.6 2DG treatment group, there were no differences in percentage of detached cells or total cell number before and after clonal selection, aside from a slight increase in the percentage of dead cells in the adapted clones. In contrast, following 4.8 2DG treatment there was an increase in the percentage of detached cells (ns) and a significant increase in total cell number (p<0.01), with no differences in the percentage of dead cells in the adapted clones. Interestingly, CLONE^ctrl^, derived through selection of detached cells in control (cells without treatment) displayed enhanced proliferation compared to untreated cells, with a modest increase in the percentage of detached cells and no significant difference in cell death.

We further examined the effects of clonal selection on cell viability and total cell number following reattachment. No significant differences in the percentage of dead cells were observed in CLONE^ctrl^, CLONE^2DG^ and CLONE^polyHEMA^ compared to ctrl (**Figures 2E, G**). However, CLONE^Met+2DG^ showed a modest but significant increase in cell death (12%, p<0.05, **Figure 2E**), indicating that the detached clones largely remain viable after reattachment. Proliferation analysis after reattachment revealed a slight reduction in CLONE^ctrl^ and CLONE^2DG^ compared to ctrl. Notably, CLONE^Met+2DG^ had proliferation rate less than 50% of the ctrl rate (ns), which agrees with the increased percentage of dead cells after reattachment (**Figure 2G**). In contrast, CLONE^polyHEMA^ exhibited viability and proliferation comparable to ctrl, suggesting that detachment *per se* does not inherently impair the ability of cells to proliferate once reattached.

Altogether, these results indicate that the selection process yielded cell clones with a non-significantly increased detachment rate, without a corresponding rise in the percentage of dead cells within the detached population. This suggests that the selected clones are better adapted to growth under anchorage-independent conditions. Furthermore, upon reattachment, most clones were able to regain their proliferative capacity. An exception was CLONE^Met+2DG^, which exhibited a slower proliferation rate (ns) and a modest increase in cell death, indicating partial impairment in recovery following reattachment.

### 3.2 Transcriptional diversity between wild-type cells and clones obtained through repeated selection of detached cells

**Figure 3:**
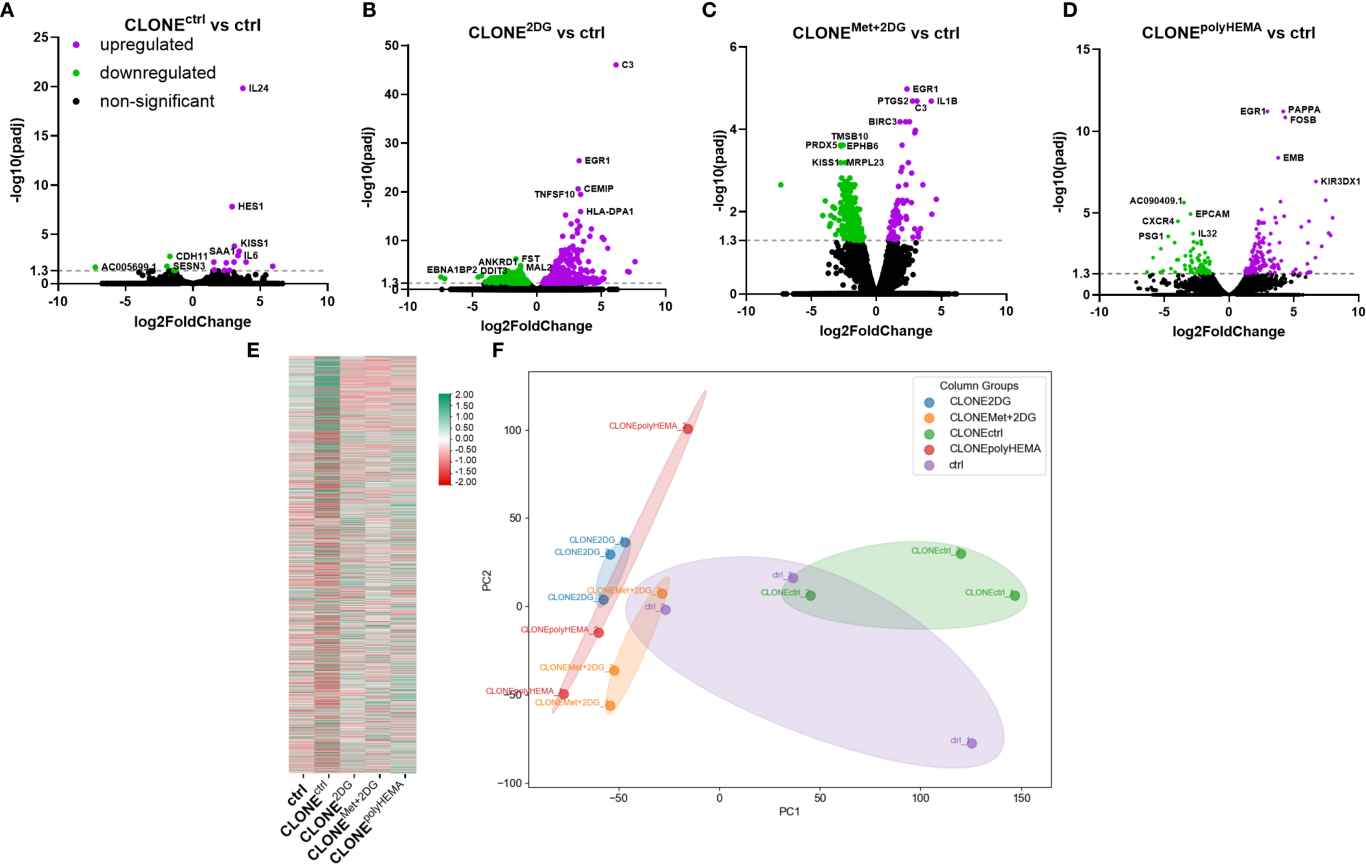
Transcriptomic analysis of differential gene expression in the selected clones. MDA-MB-231 cells were grown in RPMI 1640 media with 1 g/L glucose and treated with 2-deoxy-D-glucose alone or in combination with metformin or grown on polyHEMA coated plates. After 72 hours, detached cells were reseeded in compound-free media for 72h. Reattached cells were reseeded again and treated with the same compounds. We have performed 4 cycles of detached cell selection. After the last cycle, detached cells were reseeded in compound free media and left to proliferate for 72 hours. RNA expression levels of reattached clones were determined with RNA sequencing. **(A–D)** Volcano plot of differentially expressed genes in CLONE^ctrl^ **(A)**, CLONE^2DG^ **(B)**, CLONE^Met+2DG^ **(C)** or CLONE^polyHEMA^ **(D)** versus ctrl. **(E)** Heatmap of 10–000 differentially expressed genes. **(F)** PCA analysis of total RNA transcriptomics. The mean ± SEM for three independent experiments is shown. Normalized Z score values for three independent experiments are shown in heatmap. Met, metformin; 2DG, 2-deoxy-D-glucose.

To determine whether our adapted clones exhibit unique transcriptomic programs, that enable them to overcome anoikis, reattach, and proliferate in new environments, we conducted RNA sequencing (RNAseq) and compared gene expression profiles of selected clones to control cells. Differential gene expression analysis (DESeq2 p-value ≤ 0.05 |log2FoldChange|≥0.0) revealed several statistically significant upregulated and downregulated genes in the adapted clones. CLONE^ctrl^ showed 13 upregulated and 5 downregulated genes compared to ctrl (**Figure 3A**). CLONE^2DG^ had 599 upregulated and 955 downregulated genes compared to ctrl (**Figure 3B**). CLONE^Met+2DG^ showed 84 upregulated and 448 downregulated genes compared to ctrl (**Figure 3C**). CLONE^polyHEMA^ had 133 upregulated and 84 downregulated genes compared to ctrl (**Figure 3D**). One of the most significantly upregulated genes in CLONE^2DG^, CLONE^Met+2DG^ and CLONE^polyHEMA^ versus ctrl, was *EGR1*, a transcriptional regulator involved in differentiation and mitogenesis. In CLONE^2DG^ (compared to ctrl) other significantly upregulated genes were either connected to EMT, tumor cell growth, cell adhesion, motility, such as *CEMIP* and *FN1*, and apoptosis, such as *TNFSF10* and *BIRC3*. Interestingly, some genes were also connected to the immune response, especially the activation of the complement, such as *C3*, *FN1*, *HLA-DPA1*, *BIRC3* and *C1S*. In CLONE^Met+2DG^ versus ctrl, the most significantly upregulated genes were related to transcription regulation, such as *EGR1, FOSB* and *HES*. VEGF production through *IL1B* was also upregulated, as well as metabolism of various substrates, including fatty acids (*CYP1B1*). Interestingly, some genes here were also connected to the immune response, especially the activation of the complement, such as *C3*, *BIRC3* and *IL1A*. In CLONE^polyHEMA^ versus ctrl, one of the most significantly upregulated genes were cancer stemness markers *CD69* and *ROBO1*, which regulates cell migration. *FOSB*, gene transcription enhancer was also upregulated. The most significantly downregulated genes in CLONE^2DG^ versus ctrl were activation of endothelial cell, *ANKRD1*, transcription factor in ER stress response *DDIT3* and polarized transport *MAL2*. In CLONE^Met+2DG^ versus ctrl, the most downregulated genes were related to mitochondria, such as *MRPL23* (mitochondrial ribosome) and *COX5B*, component of the cytochrome C oxidase. Interestingly, peroxidase *PRDX5* and metastasis suppressor protein *KISS1* were also downregulated. In CLONE^polyHEMA^ versus ctrl, one of the most downregulated genes was *TSPAN8*, which plays a role in the regulation of cell development, activation, growth and motility, additionally epithelial cell adhesion molecule (*EPCAM*) was also downregulated.

To further analyze the differences between the wild-type, control cells, and the selected clones, we analyzed the expression of differentially expressed genes within KEGG pathways (**Figure S2**). The clones derived from repeated selection of detached cells treated with either 4.8 2DG (CLONE^2DG^) or 0.6 2DG + Met (CLONE^Met+2DG^) showed enrichment in the same key pathways compared to ctrl: oxidative phosphorylation, carcinogenesis due to ROS and pathways associated with Parkinson and prion disease (**Supplementary Figures S2B, C**). Interestingly, CLONE^polyHEMA^, derived from repeated selection of cells grown on polyHEMA-coated plates, also showed enrichment in these same pathways compared to ctrl (**Supplementary Figure S2D**). In contrast, CLONE^ctrl^ (versus control) exhibited significant differential expression in pathways related to NF-κB and TNFα signaling (**Supplementary Figure S2A**).

Gene Ontology (GO) term analysis of the differentially expressed genes revealed that the most enriched pathways in both CLONE^2DG^ and CLONE^Met+2DG^ (compared to ctrl) were associated with protein translation, the endoplasmic reticulum (ER) and mitochondrial function (**Supplementary Figures S3B, C**). In contrast, the adapted clone CLONE^ctrl^ showed enrichment in pathways related to ER stress, hypoxia, receptor and cytokine activity, and cell-cell adhesion (**Supplementary Figure S3A**). In the CLONE^polyHEMA^ sample (compared to ctrl), the most enriched pathways were related to mitochondrial function and respiration, as well as protein targeting in ER (**Supplementary Figure S3D**). A representative heatmap of differentially expressed genes is shown in **Figure 3E**. Principal component analysis (PCA) revealed that CLONE^ctrl^ clustered closely with control cells, while CLONE^2DG^ and CLONE^polyHEMA^ showed a similar transcriptional profile, clustering together (**Figure 3F**).

### 3.3 Adapted clones have upregulated mesenchymal markers

**Figure 4:**
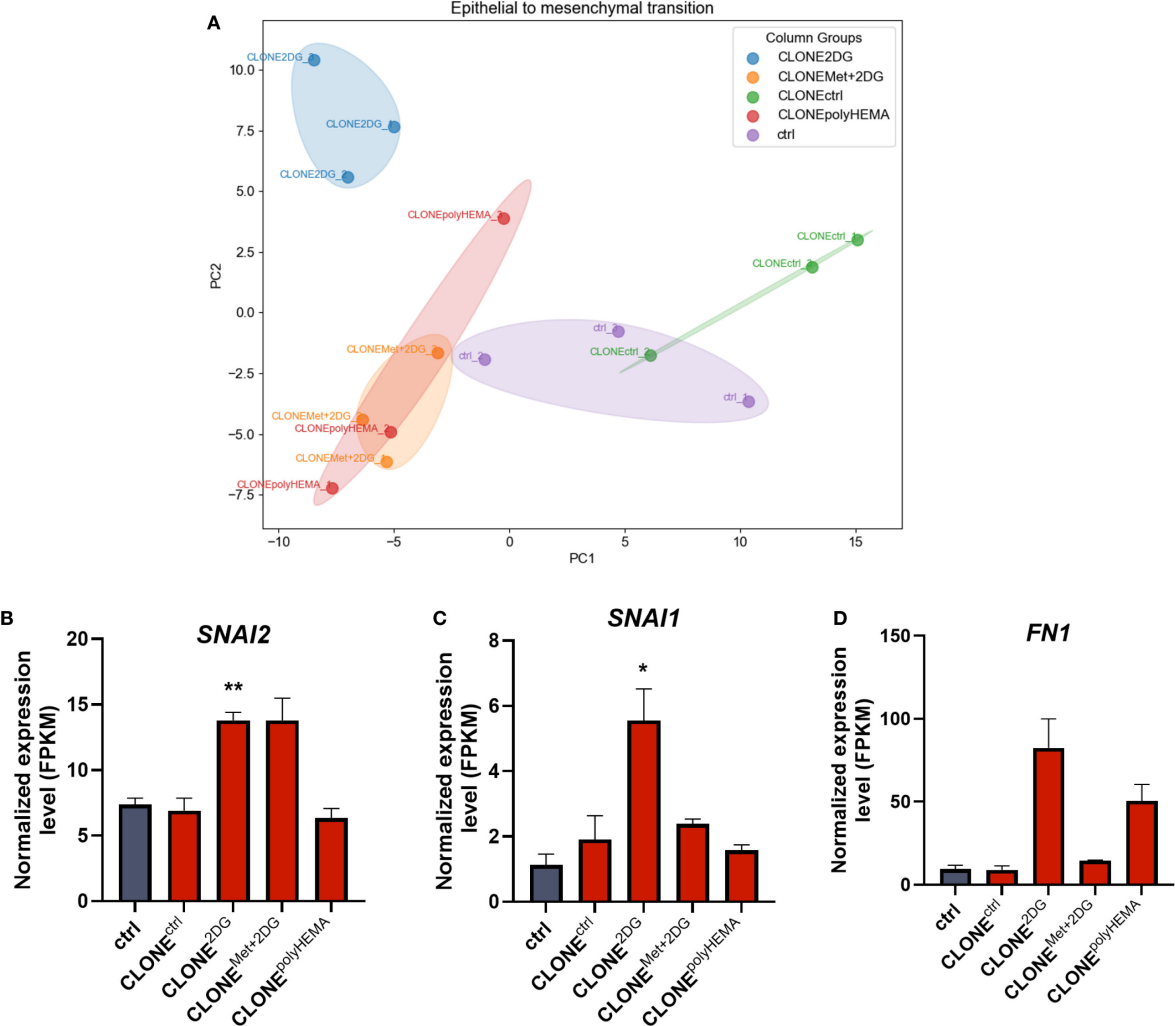
Transcriptomic analysis of EMT. MDA-MB-231 cells were grown in RPMI 1640 media with 1 g/L glucose and treated with 2-deoxy-D-glucose alone or in combination with metformin or grown on polyHEMA coated plates. After 72 hours, detached cells were reseeded in compound-free media for 72h. Reattached cells were reseeded again and treated with the same compounds. We have performed 4 cycles of detached cell selection. After the last cycle, detached cells were reseeded in compound free media and left to proliferate for 72 hours. RNA expression levels of reattached cells were determined with RNA sequencing. PCA analysis of EMT **(A)**. Main genes in EMT pathway are shown in more detail **(B–D)**. The mean ± SEM for three independent experiments is shown. *p<0.05, **p<0.01, ***p<0.001, ****p<0.0001 versus attached control cells as determined by one-way ANOVA with Dunnett’s *post hoc* test. FPKM, fragments per kilobase of transcript per million mapped reads; Met, metformin; 2DG, 2-deoxy-D-glucose.

Epithelial to mesenchymal transition (EMT) and its reversal process, mesenchymal to epithelial transition (MET), are key prerequisites for cancer metastasis. Transcriptomic analysis showed differential expression of several genes involved in EMT (**Supplementary Figure S4A**). PCA analysis showed that all selected treated clones clustered separately from ctrl, with CLONE^polyHEMA^ and CLONE^Met+2DG^ clustered closely, whereas CLONE^2DG^ clustered completely separately, indicating the most distinct gene expression profile related to EMT (**Figure 4A**). Specifically, CLONE^2DG^ exhibited upregulation of mesenchymal markers *SNAI1* (p<0.05), *SNAI2* (p<0.05), and *FN1* (ns) (**Figures 4B–D**). In contrast, CLONE^Met+2DG^ showed only upregulation of *SNAI2* (ns) and CLONE^polyHEMA^ only of *FN1* (ns). Overall, a similar trend in EMT gene expression was observed across CLONE^2DG^, CLONE^Met+2DG^ and CLONE^polyHEMA^ (**Supplementary Figure S4A**).

Altogether, these results suggest that the selection of detached cells leads to the emergence of clones with enhanced expression of mesenchymal markers and reduced expression of epithelial markers. This gene expression profile aligns with the observed increase in the proportion of detached cells, indicating a greater propensity of these cells to lose adhesion and detach from the surface.

### 3.4 Cell stress response to ER stress and reactive oxygen species

**Figure 5:**
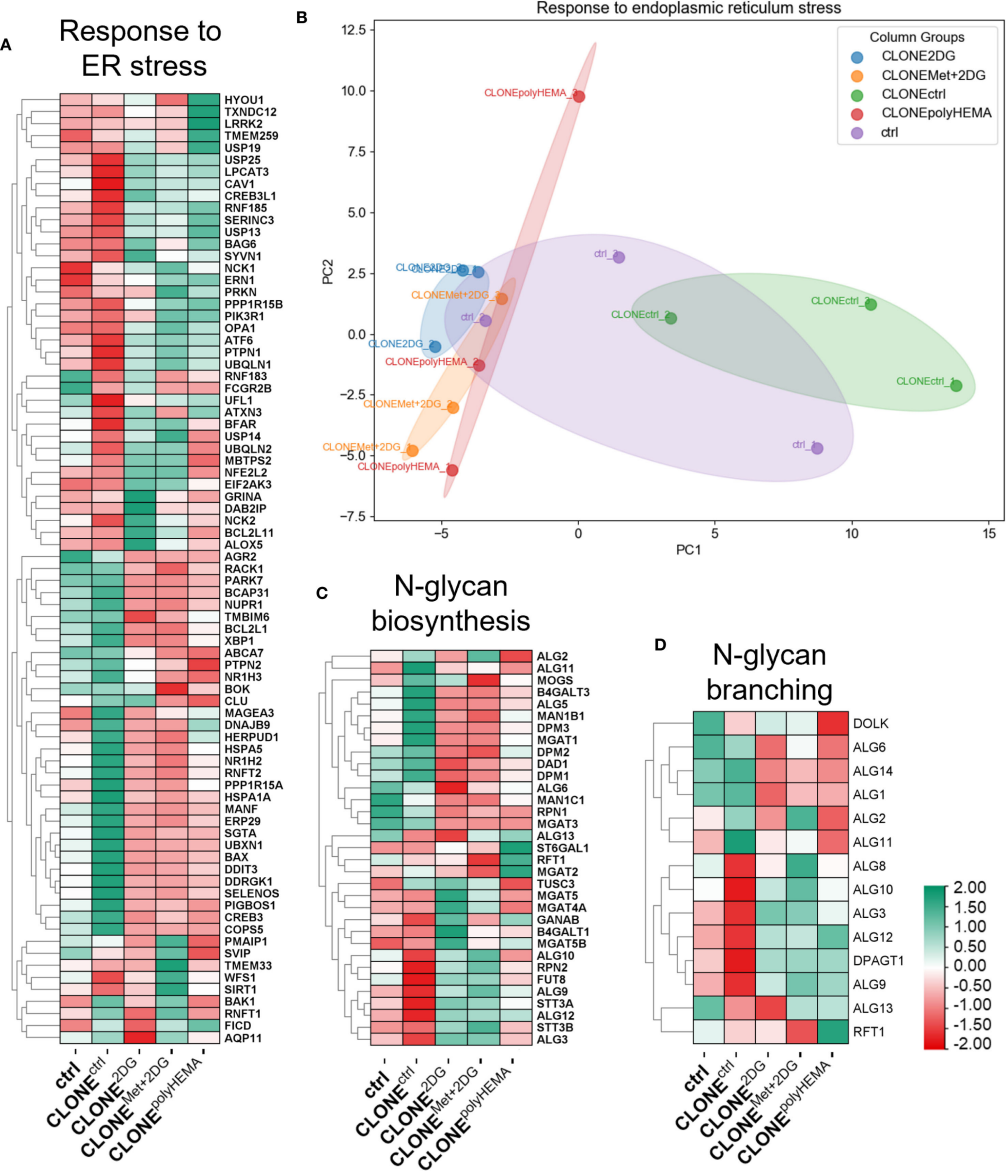
Transcriptomic analysis of ER stress and N-glycan biosynthesis. MDA-MB-231 cells were grown in RPMI 1640 media with 1 g/L glucose and treated with 2-deoxy-D-glucose alone or in combination with metformin or grown on polyHEMA coated plates. After 72 hours, detached cells were reseeded in compound-free media for 72h. Reattached cells were reseeded again and treated with the same compounds. We have performed 4 cycles of detached cell selection. After the last cycle, detached cells were reseeded in compound free media and left to proliferate for 72 hours. RNA expression levels of reattached cells were determined with RNA sequencing. Heatmap of genes involved in ER stress response **(A)**, N-glycan biosynthesis **(C)**, and N-glycan branching **(D)**. PCA analysis of response to ER stress **(B)**. The mean ± SEM for three independent experiments is shown. Normalized Z score values for three independent experiments are shown in heatmap. Met, metformin; 2DG, 2-deoxy-D-glucose.

We have previously demonstrated that cell detachment is triggered by inhibition of protein N-glycosylation, accompanied with ER stress and activation of unfolded protein response (UPR) (70). To investigate whether UPR and ER stress response are reversible upon cell reattachment and to assess differences between adapted clones and ctrl cells, we analyzed relevant markers in both conditions (**Figures 5**, **S5**). Additionally, we have analyzed how non-selected wild-type cells respond to ER stress during treatment with 2DG and Met+2DG, to understand how adaptations differ in the absence of selection pressure.

We have observed a similar gene expression profile related to UPR and ER stress in ctrl and CLONE^ctrl^ cells, although CLONE^ctrl^ exhibited more pronounced up- or downregulation of several genes compared to ctrl (**Figures 5A, B**, **S5A, B**). PCA analysis of UPR showed that CLONE^2DG^, CLONE^Met+2DG^ and CLONE^polyHEMA^ clustered together, while CLONE^ctrl^ clustered in the opposite direction and ctrl cells occupied an intermediate position (**Figure 5B**). CLONE^2DG^, CLONE^Met+2DG^ and CLONE^polyHEMA^ displayed a trend of upregulated *ATF6* (ns) and downregulated *BAX, ATF4, HSPA5* (all ns), and *DDIT3* (p<0.05 in CLONE^polyHEMA^), while there were no differences in the expression of other UPR-related genes (**Supplementary Figure S5**).

The expression of genes involved in N-glycan biosynthesis (**Figures 5C, D**), showed a similar expression patterns to that of the UPR and ER stress response, with CLONE^2DG^ and CLONE^Met+2DG^ having expectedly similar pattern due to effects of 2DG or Met+2DG treatments on N-glycosylation. Furthermore, we have observed a similar trend in expression of genes involved in oligosaccharyltransferase (OST) complex and N-glycan branching pathway, where CLONE^2DG^, CLONE^Met+2DG^ and CLONE^polyHEMA^ showed complete opposite gene expression compared to ctrl (**Figures 5D**, **S6**). KEGG pathway analysis showed decreased expression of various genes across N-glycan biosynthesis for CLONE^2DG^, CLONE^Met+2DG^ and CLONE^polyHEMA^ compared to ctrl (**Supplementary Figure S7**). Altogether these results suggest that UPR and impaired protein N-glycosylation induced by cell detachment are not fully reversible upon cell reattachment. It is plausible that cells require additional time post-reattachment to fully resolve UPR and restore homeostasis.

**Figure 6:**
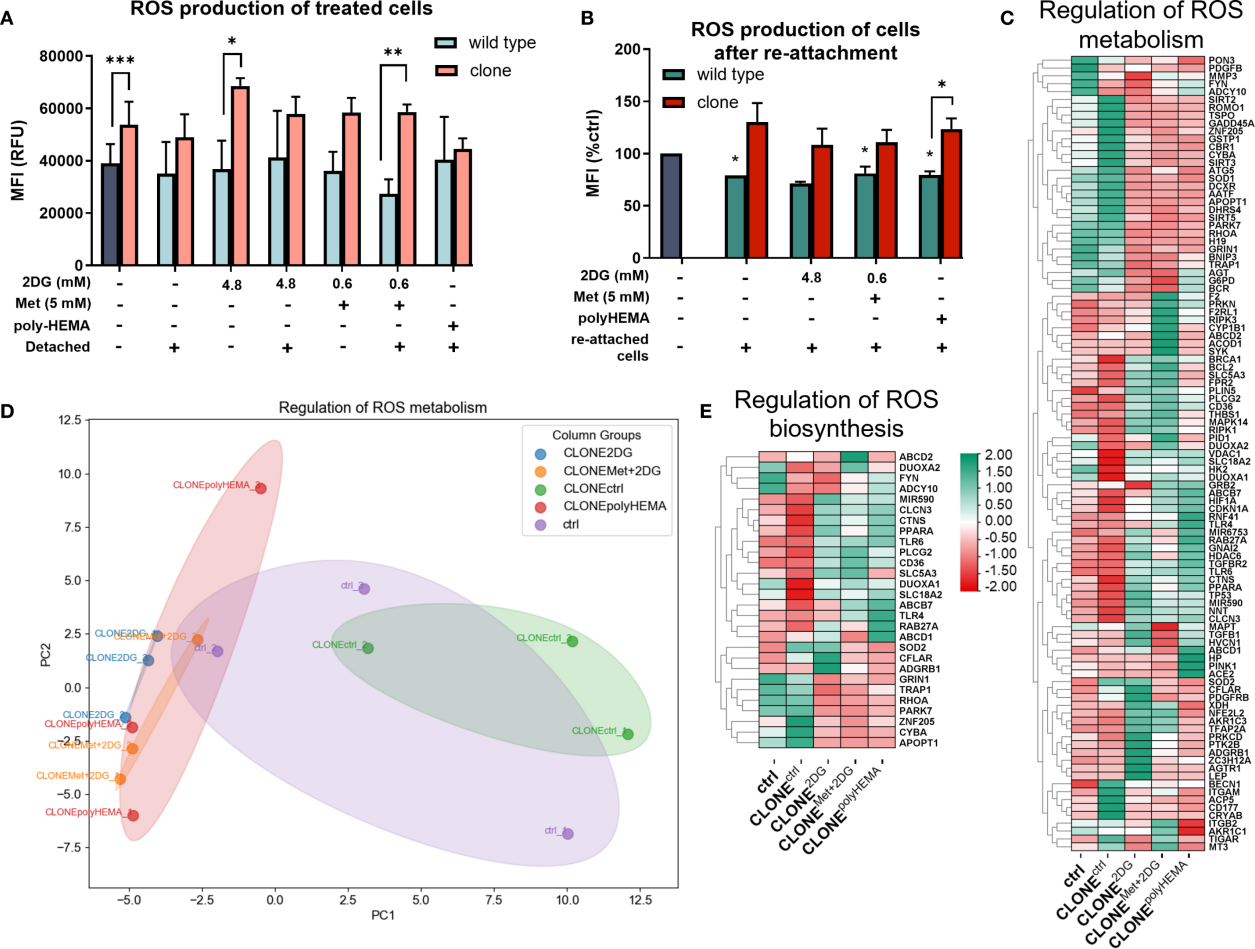
ROS metabolism in adapted clones of MDA-MB-231 cells during and after treatments. MDA-MB-231 cells were grown in RPMI 1640 media with 1 g/L glucose and treated with 2-deoxy-D-glucose alone or in combination with metformin or grown on polyHEMA coated plates. After 72 hours, detached cells were reseeded in compound-free media for 72h. Reattached cells were reseeded again and treated with the same compounds. We have performed 4 cycles of detached cell selection. After the last treatment, detached and attached cells were collected separately and reactive oxygen species (ROS) production **(A)** was determined with flow cytometry. After the last cycle, detached cells were also reseeded in compound free media and left to proliferate for 72 hours, and the re-attached cells were collected where ROS were again analyzed with flow cytometry **(B)**. RNA expression levels of reattached cells were determined with RNA sequencing. **(C, E)** Heatmap of genes involved in regulation of ROS metabolism **(C)** and regulation of ROS biosynthesis **(E)**. **(D)** PCA analysis of regulation of ROS metabolism. The mean ± SEM for three independent experiments is shown. Normalized Z score values for three independent experiments are shown in heatmap. *p<0.05, **p<0.01, ***p<0.001, ****p<0.0001 versus attached control cells as determined by one-way ANOVA with Dunnett’s *post hoc* test. MFI, mean fluorescence intensity; RFU, relative fluorescence unit; Met, metformin; 2DG, 2-deoxy-D-glucose.

Previous studies have demonstrated that pathways regulating ROS are altered in cells growing under anchorage-independent conditions (reviewed in (72)), see also (47, 73)). Based on this, we hypothesized that ROS-related gene expression would also be altered in the adapted clones following repeated growth in a detached state. First, we analyzed cellular ROS production during the treatment with 2DG or Met+2DG in selected clones. Notably, CLONE^Met+2DG^ treated with 5 Met + 0.6 2DG showed a statistically significant increase in ROS (p<0.05) in both attached and detached cells, compared to attached control (ctrl) (**Figure 6A**). In the attached, treated clones we observed a significant increase in ROS compared to the wild-type cells treated with the same compounds: CLONE^ctrl^ (p<0.001), CLONE^2DG^ (p<0.05) and CLONE^Met+2DG^ (p<0.01) (**Figure 6A**). We then assessed cellular ROS levels in reattached adapted clones (**Figure 6B**). In all reattached wild-type cells, ROS levels were significantly reduced compared to ctrl (p<0.05 for WT^ctrl^, WT^Met+2DG^ and WT^polyHEMA^), while in the clones, ROS levels remained slightly elevated compared to ctrl (ns) (**Figure 6B**). Notably, CLONE^polyHEMA^ had significantly higher ROS levels than WT^polyHEMA^ (p<0.05). Transcriptomic analysis showed altered expression of genes involved in ROS production and metabolism in the selected clones (**Figures 6C–E**). PCA analysis showed strong similarities among CLONE^2DG^, CLONE^Met+2DG^ and CLONE^polyHEMA^, with CLONE^ctrl^ clustered distinctly apart (**Figure 6D**). We observed a trend of downregulation of ROS-scavenging enzymes such as *GPX1* (ns) and *SOD1* (ns) in CLONE^2DG^, CLONE^Met+2DG^ and CLONE^polyHEMA^, while the expression levels of *CAT* and *NO*X genes remained unchanged (**Supplementary Figure S8**). Overall, these findings indicate that the adapted clones exhibit elevated cellular ROS levels following four cycles of selection under anchorage-independent conditions. The downregulation of key ROS scavengers may explain why ROS levels fail to return to baseline after cell reattachment.

**Figure 7:**
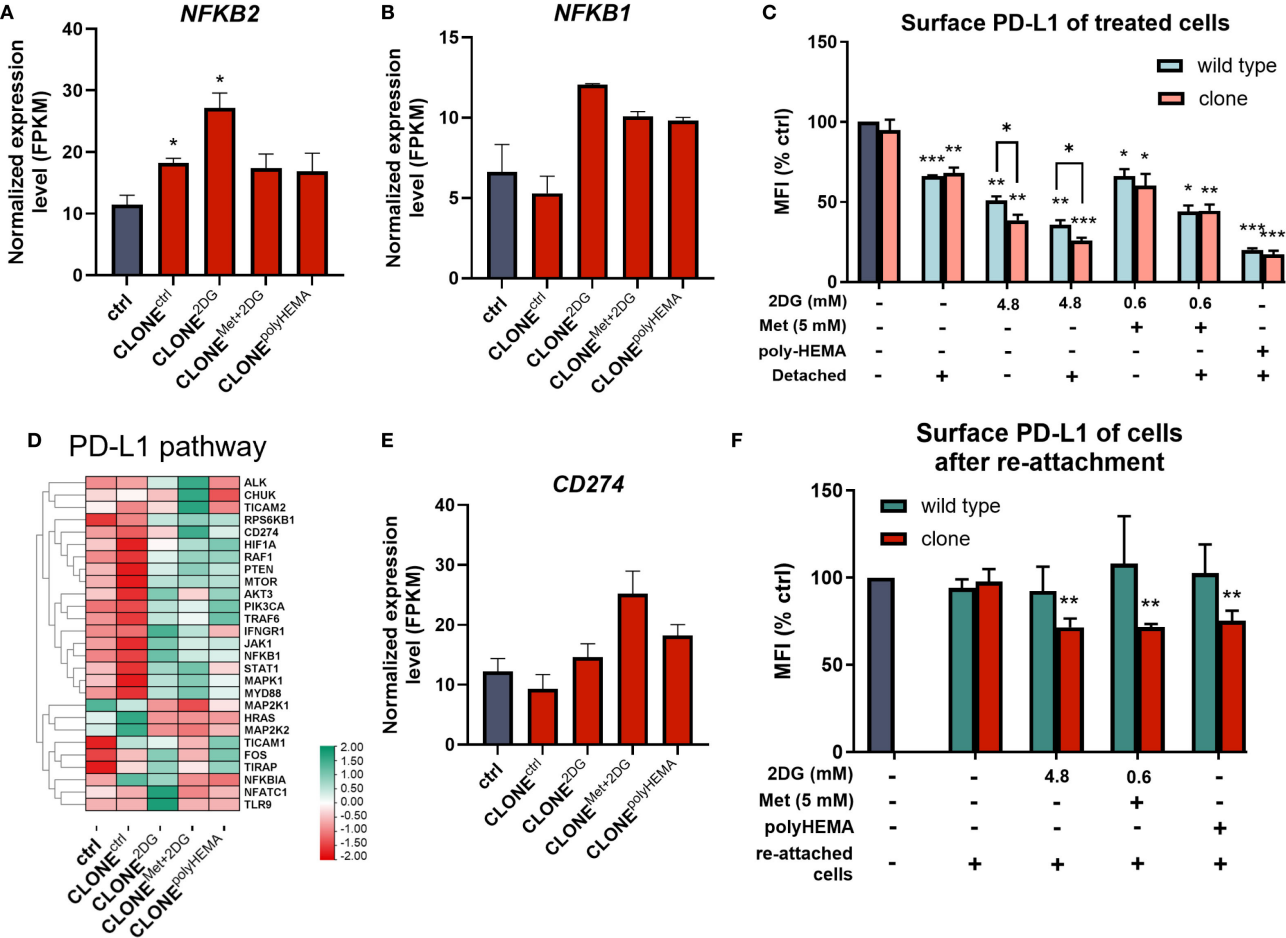
Relation between NF-κB and PD-L1 pathways in the adapted clones during and after treatments. MDA-MB-231 cells were grown in RPMI 1640 media with 1 g/L glucose and treated with 2-deoxy-D-glucose alone or in combination with metformin or grown on polyHEMA coated plates. After 72 hours, detached cells were reseeded in compound-free media for 72h. Reattached cells were reseeded again and treated with the same compounds. We have performed 4 cycles of detached cell selection. After the last treatment, detached and attached cells were collected separately and surface PD-L1 **(C)** was determined with flow cytometry, the mean fluorescence intensity (MFI) is shown as percentage of control. After the last cycle, detached cells were also reseeded in compound free media and left to proliferate for 72 hours, and the re-attached cells were also collected where surface PD-L1 was again analyzed with flow cytometry **(F)**, MFI is shown as percentage of control. Heatmap of genes involved in PD-L1 pathway **(D)**. RNA expression levels of reattached cells were determined with RNA sequencing. Main genes in NF-κB **(A, B)** and PD-L1 expression **(E)** are shown in more detail. The mean ± SEM for three independent experiments is shown. Normalized Z score values for three independent experiments are shown in heatmap. *p<0.05, **p<0.01, ***p<0.001, ****p<0.0001 versus attached control cells as determined by one-way ANOVA with Dunnett’s *post hoc* test. FPKM - fragments per kilobase of transcript per million mapped reads; MFI, mean fluorescence intensity; Met, metformin; 2DG, 2-deoxy-D-glucose.

ROS are known to induce the expression of NF-κB, a key transcription factor that regulates immune response and serves as a marker for proinflammation and carcinogenesis. In our adapted clones, we observed an increasing trend in the expression of *NFKB2* (p<0.05) and *NFKB1* (ns) compared to ctrl (**Figures 7A, B**). Additionally, genes involved in the canonical NF-κB pathway were differentially expressed in CLONE^2DG^, CLONE^Met+2DG^ and CLONE^polyHEMA^ (**Supplementary Figure S9**). *STUB1*, which promotes the ubiquitination and degradation of p65 (subunit of NF-κB), showed a trend of downregulation in CLONE^2DG^, CLONE^Met+2DG^ and CLONE^polyHEMA^ (all ns) (**Supplementary Figure S9B**). Furthermore, *STUB1* is also known to promote PD-L1 ubiquitination and degradation. A similar pattern was observed with *CSN5*, a subunit of CSN complex, that regulates NF‐κB by deubiquitinating IκBα and also stabilizes PD-L1 expression in breast cancer, with a significant upregulation in CLONE^ctrl^ (p<0.05) (**Supplementary Figure S9C**). Taken together, these findings suggest that NF-κB transcription is upregulated and appears to be protected from degradation.

Activation of NF-κB is, beside MAPK and PI3K/Akt signaling pathways, as well as various cytokines and miRNAs, known to induce *CD274* transcription (codes PD-L1) (74). In our study, we observed significantly reduced surface PD-L1 expression across all treated clones (p<0.05), with more pronounced downregulation in the adapted clones compared to treated wild-type cells, especially in 4.8 2DG treatment (p<0.05 in both attached and detached treated cells) (**Figure 7C**). Interestingly, *CD274* transcription showed an increasing trend in CLONE^Met+2DG^ (ns) compared to ctrl (**Figure 7E**). Among the clones, CLONE^ctrl^ showed the fewest changes in PD-L1 related gene expression, while CLONE^2DG^, CLONE^Met+2DG^ and CLONE^polyHEMA^ seemed to have upregulated multiple pathways associated with PD-L1 expression (**Figure 7D**). Despite increased *CD274* transcription, surface PD-L1 expression was downregulated in re-attached CLONE^2DG^ (p<0.01), CLONE^Met+2DG^ (p<0.01) and CLONE^polyHEMA^ (p<0.01), compared to ctrl (**Figure 7F**). In contrast, surface PD-L1 levels in wild-type cells returned to baseline. These results suggest that repeated treatments with 2DG or Met+2DG may lead to a lasting reduction in surface PD-L1 expression. This observation aligns with previous findings (51, 70), which demonstrated that N-glycosylation inhibition and ER stress can reduce surface PD-L1 expression. Non-normalized values and gating strategy for PD-L1 are shown in **Supplementary Figure S10**. KEGG pathway analysis showed consistent upregulation of genes involved in PD-L1 expression for CLONE^2DG^, CLONE^Met+2DG^ and CLONE^polyHEMA^ compared to ctrl (**Supplementary Figure S11**).

Interestingly, we also observed a pattern of increased expression of *ADAM17* in CLONE^Met+2DG^, CLONE^2DG^and CLONE^polyHEMA^ (all ns), as well as increased *ADAM10* in CLONE^2DG^ (ns) (**Supplementary Figures S10D, E**). These ADAM proteases are known to cleave PD-L1 from the surface of malignant cells and extracellular vesicles (75, 76).

Taken together, our findings indicate that repeated selection of detached cells treated with 2DG or Met+2DG leads to elevated ROS levels, which in turn induce NF-κB activation and promote *CD274* (PD-L1) transcription. However, despite increased transcription, surface PD-L1 levels were decreased, indicating the involvement of posttranscriptional regulatory mechanisms such as N-glycosylation.

### 3.5 Reattached adapted clones have upregulated stemness markers and exhibit senescent-like phenotype

**Figure 8:**
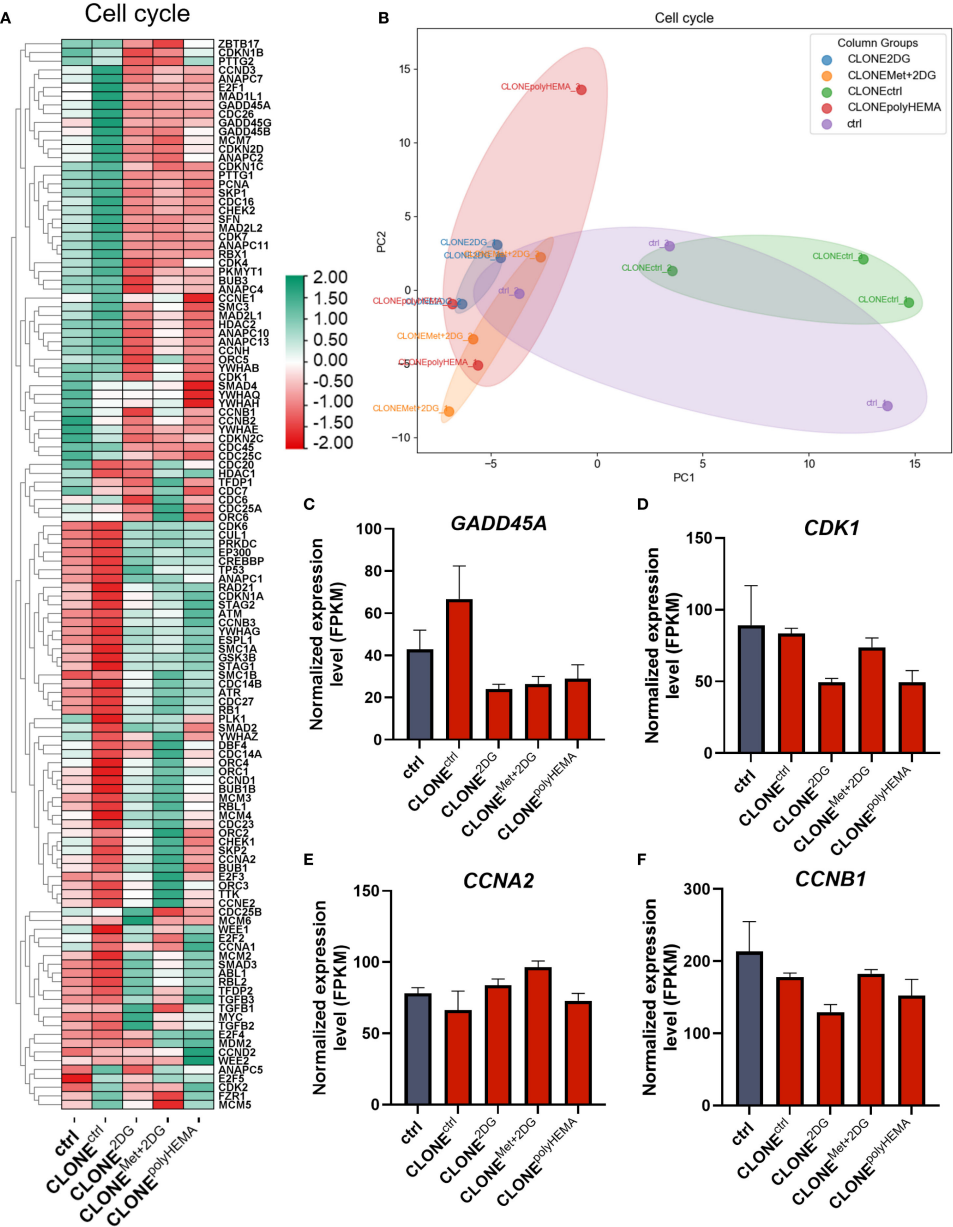
Transcriptomic analysis of cell cycle. MDA-MB-231 cells were grown in RPMI 1640 media with 1 g/L glucose and treated with 2-deoxy-D-glucose alone or in combination with metformin or grown on polyHEMA coated plates. After 72 hours, detached cells were reseeded in compound-free media for 72h. Reattached cells were reseeded again and treated with the same compounds. We have performed 4 cycles of detached cell selection. After the last cycle, detached cells were reseeded in compound free media and left to proliferate for 72 hours. RNA expression levels of reattached cells were determined with RNA sequencing. **(A)** Heatmap of genes involved in cell cycle pathway. **(B)** PCA analysis of cell cycle. Main genes in cell cycle regulation **(C–F)** are shown in more detail. The mean ± SEM for three independent experiments is shown. Normalized Z score values for three independent experiments are shown in heatmap. *p<0.05, **p<0.01, ***p<0.001, ****p<0.0001 versus attached control cells as determined by one-way ANOVA with Dunnett’s *post hoc* test. FPKM, fragments per kilobase of transcript per million mapped reads; Met, metformin; 2DG - 2-deoxy-D-glucose.

Deregulation of the cell cycle is one of the hallmarks of cancer progression. While the cell cycle is typically upregulated during tumor development, it becomes inhibited when cells detach from the primary tumor. In our study, we observed decreased total cell number after cell reattachment suggesting decrease in proliferation, despite having differential expression of genes involved in the cell cycle in CLONE^2DG^, CLONE^Met+2DG^ and CLONE^polyHEMA^ compared to ctrl (**Figures 8A, B**). Furthermore, cell cycle arrest inhibitors *GADD45A* and cell cycle regulator *CDK1* showed a trend of downregulation, while tumor suppressor *RB1* was upregulated (ns) in the same clones compared to ctrl (**Figures 8C, D**, **S12A**). Cell viability analysis following reattachment showed a significant increase in the percentage of dead cells only in CLONE^Met+2DG^ (p<0.05), while no significant differences were observed in other adapted clones. PCA analysis showed similar clustering of CLONE^2DG^, CLONE^Met+2DG^ and CLONE^polyHEMA^ and distinct from ctrl (**Figure 8B**).

We next analyzed the expression of cyclins and cyclin-dependent kinases (CDKs). A slight decrease in *CDK1* expression was observed in CLONE^2DG^ and CLONE^polyHEMA^ (ns for both), which may affect cell cycle progression, as *CDK1* leads to cell cycle progression when bound to its cyclin partner Cyclin A or Cyclin B (**Figure 8D**). While *CCNA2* (cyclin A) expression remained unchanged in all adapted clones, *CCNB1* (cyclin B) showed a trend of downregulation in CLONE^2DG^ and CLONE^polyHEMA^ (ns) (**Figures 8E, F**). Expression of *CDK2* and *CDK4* were also unchanged across all clones (**Supplementary Figures S12B, C**). Similarly, *CCNE1* (cyclin E), which partners with CDK2, was also unchanged in adapted clones (**Supplementary Figures S12E**). In contrast, *CCND1* (Cyclin D1) a key regulator of cell cycle progression, exhibited a slight trend of upregulation in CLONE^Met+2DG^ (ns), as well as its binding kinase *CDK6* (**Supplementary Figures S12D, F**). Despite some alteration in transcription in cell cycle progression, this was not reflected on the total cell number after reattachment. In fact, CLONE^2DG^ and CLONE^Met+2DG^ had reduced total cell number compared to ctrl (**Figure 2G**) indicated reduced proliferation. These findings suggest that while cell cycle-related gene expression shows a trend of upregulation, actual proliferation is not correspondingly increased after reattachment.

**Figure 9:**
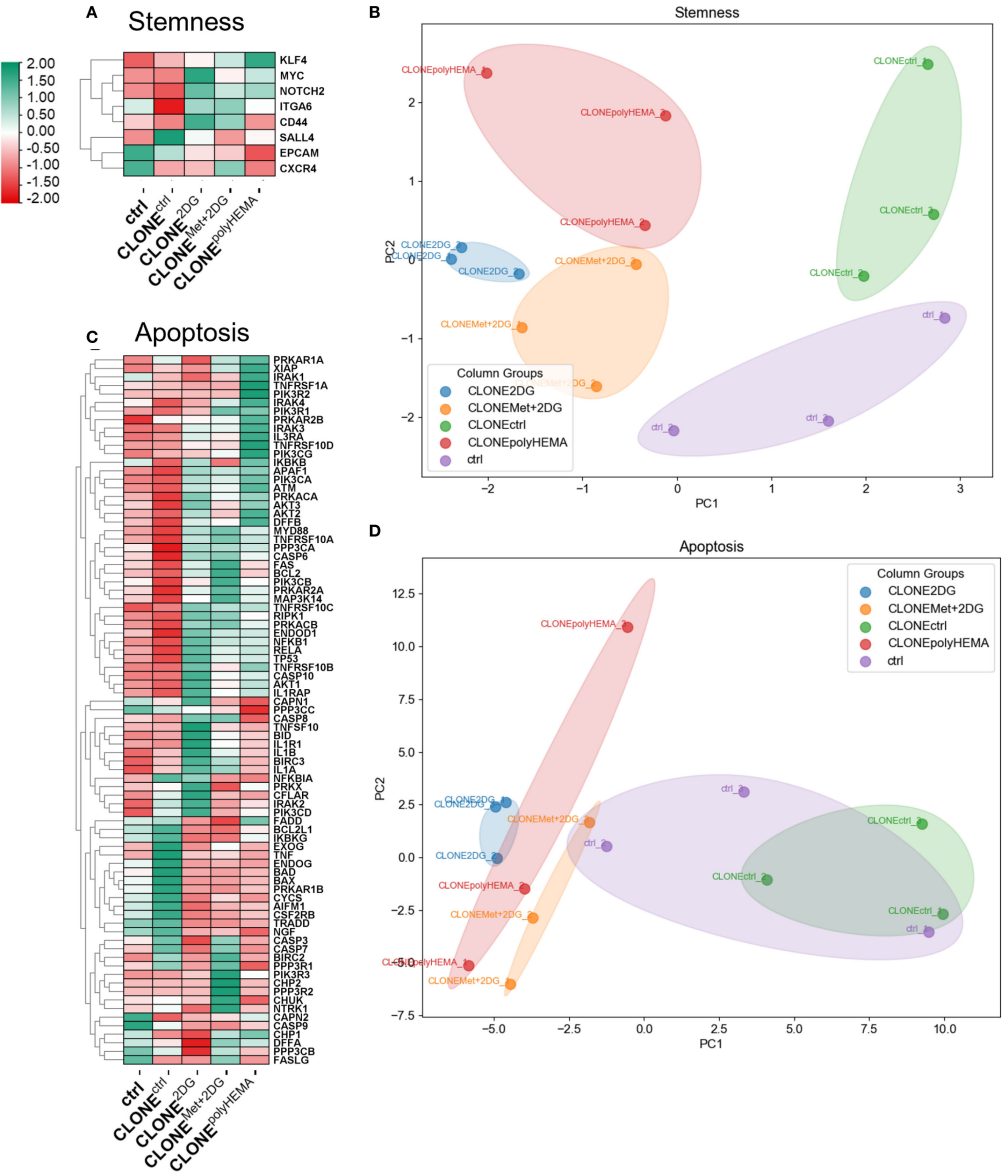
Transcriptomic analysis of apoptosis and stemness. MDA-MB-231 cells were grown in RPMI 1640 media with 1 g/L glucose and treated with 2-deoxy-D-glucose alone or in combination with metformin or grown on polyHEMA coated plates. After 72 hours, detached cells were reseeded in compound-free media for 72h. Reattached cells were reseeded again and treated with the same compounds. We have performed 4 cycles of detached cell selection. After the last cycle, detached cells were reseeded in compound free media and left to proliferate for 72 hours. RNA expression levels of reattached cells were determined with RNA sequencing. **(A, C)** Heatmap of genes involved in stemness **(A)** and apoptosis **(C)** pathway. PCA analysis of stemness **(B)** and apoptosis **(D)**. The mean ± SEM for three independent experiments is shown. Normalized Z score values for three independent experiments are shown in heatmap. Met, metformin; 2DG - 2-deoxy-D-glucose.

Importantly, PCA analysis of stemness associated markers showed distinct clustering patterns among the clones: CLONE^polyHEMA^, CLONE^2DG^ and CLONE^Met+2DG^ clustered closely together and were clearly separated from CLONE^ctrl^ and ctrl (**Figure 9B**). Specifically, *MYC* (ns) displayed a trend of increase in selected clones (**Figures 9A**, **S13A**). Additionally, we identified differential expression in the apoptosis pathway in CLONE^2DG^, CLONE^Met+2DG^ and CLONE^polyHEMA^, compared to ctrl (**Figure 9C**). This included downregulation of pro-apoptotic genes such as *BAD* and *BAX*. Furthermore, *RPS6* (ribosomal protein S6), involved in protein synthesis showed a trend of downregulation in CLONE^2DG^, CLONE^Met+2DG^ and CLONE^polyHEMA^ (ns) (**Supplementary Figure S13B**), suggesting reduced translational activity. In contrast, apoptosis regulator *BCL2* showed no differential expression in obtained clones (**Supplementary Figure S13C**). PCA analysis of apoptosis-related gene expression further supported these findings, showing similar clustering of CLONE^polyHEMA^, CLONE^2DG^ and CLONE^Met+2DG^, while CLONE^ctrl^ and ctrl showed comparable expressions (**Figure 9D**). Additionally, similar clustering patterns were observed in the analysis of senescence-associated gene expression, where these three clones again showed distinct profiles compared to CLONE^ctrl^ and ctrl, which was also observed on the PCA analysis (**Supplementary Figure S14**). KEGG pathways displayed consistent upregulation of genes involved in cell senescence for CLONE^2DG^, CLONE^Met+2DG^ and CLONE^polyHEMA^, compared to ctrl (**Supplementary Figure S15**).

Taken together, these results indicate that the adapted clones exhibit a senescent-like phenotype characterized by reduced apoptosis, enhanced stemness markers and suppressed proliferation.

### 3.6 Metabolic phenotype is not altered after the repeated selection process

Functional mitochondria have previously been shown to play a key role in cell detachment, anchorage-independent growth (47, 77, 78), and metastasis formation (79). In particular, mitochondrial biogenesis and an increase in TCA cycle activity are crucial processes driving metastatic progression (80).

Given this, we investigated whether the selected adapted clones metabolically differ from wild-type cells, focusing specifically on metabolic phenotype and related pathways. We first assessed mitochondrial mass in 72-hour treated adapted clones and wild-type cells using mitotracker Orange, a dye that accumulates in the polarized, functional mitochondria. No significant differences in mitochondrial mass were observed in treated, attached wild-type cells compared to ctrl (**Supplementary Figure S16A**) as well as in the treated adapted clones– except for attached clones treated with Met + 0.6 2DG, where a slight increase was observed (ns).

**Figure 10:**
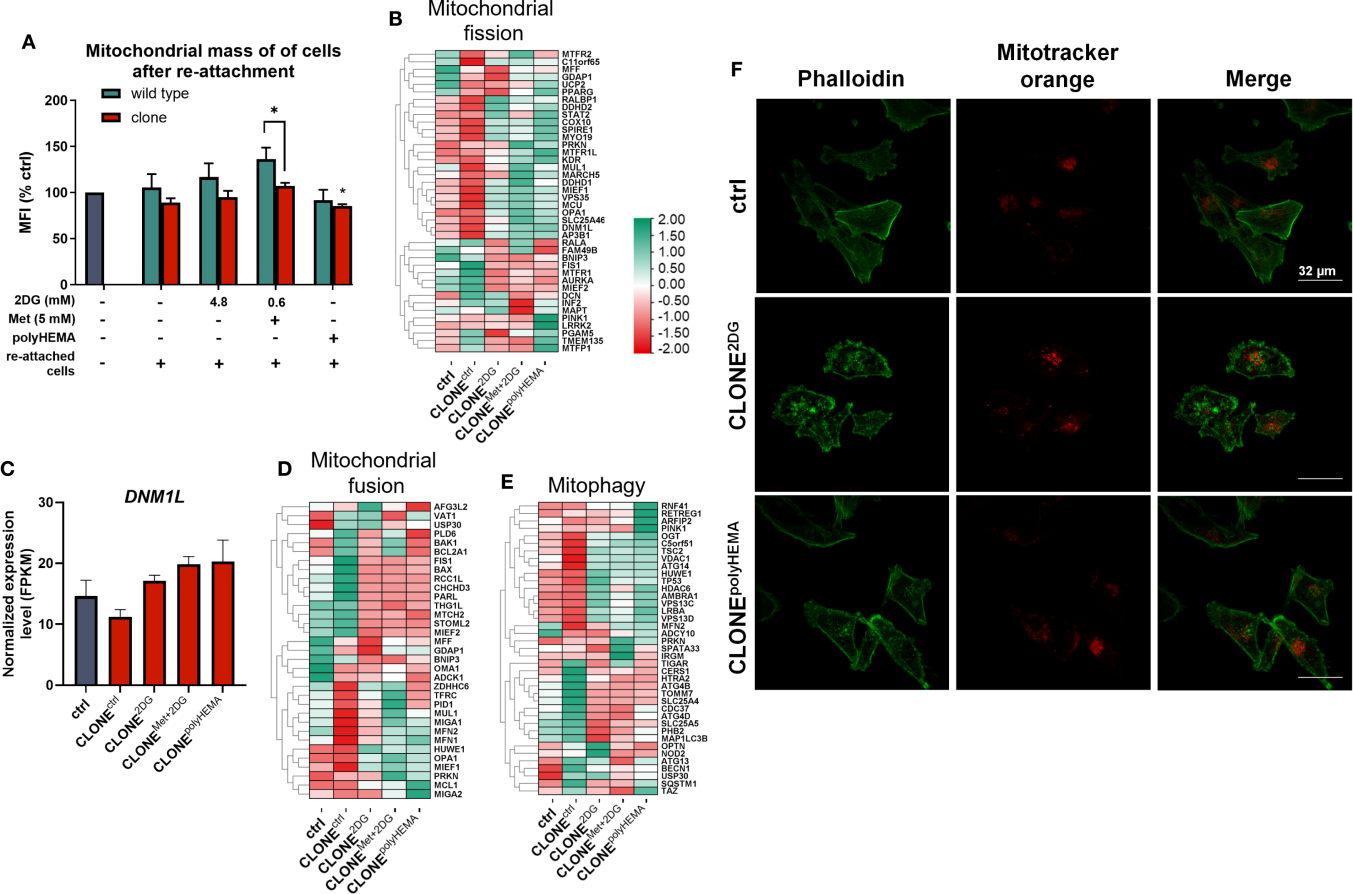
Analysis of mitochondrial dynamics. MDA-MB-231 cells were grown in RPMI 1640 media with 1 g/L glucose and treated with 2-deoxy-D-glucose alone or in combination with metformin or grown on polyHEMA coated plates. After 72 hours, detached cells were reseeded in compound-free media for 72h. Reattached cells were reseeded again and treated with the same compounds. We have performed 4 cycles of detached cell selection. After the last cycle, detached cells were reseeded in compound free media and left to proliferate for 72 hours. RNA expression levels of re-attached cells were determined with RNA sequencing. **(A)** Relative mitochondrial mass was determined by mitotracker orange dye and flow cytometry. **(B, D, E)** Heatmap of genes involved in mitochondrial fission **(B)**, mitochondrial fusion **(D)** and mitophagy **(E)**. Main gene in mitochondrial fission **(C)** is shown in more detail. Phalloidin and mitotracker orange fluorescence were detected with fluorescent microscopy **(F)**. The mean ± SEM for three independent experiments is shown. Normalized Z score values for three independent experiments are shown in heatmap. *p<0.05, **p<0.01, ***p<0.001, ****p<0.0001 versus attached control cells as determined by one-way ANOVA with Dunnett’s *post hoc* test. FPKM - fragments per kilobase of transcript per million mapped reads; Met, metformin; 2DG, 2-deoxy-D-glucose.

When analyzing re-attached cells in clones, a reduction in mitochondrial mass was observed - returning to basal levels (**Figure 10A**). CLONE^Met+2DG^ showed a significant reduction in mitochondrial mass compared to WT^Met+2DG^ (p<0.05), indicating a possible long-term impact of dual treatment on mitochondrial mass. *PPARGC1A*, a key regulator of mitochondrial biogenesis was significantly upregulated in CLONE^Met+2DG^ (p<0.05) and showed a non-significant increase in CLONE^polyHEMA^ (ns, **Supplementary Figure S16B**). However, it is important to note that *PPARGC1A* expression levels were overall very low. We also assessed genes involved in mitochondrial fission. Although *DNM1L*, a primary fission regulator, showed no significant expression changes (**Figures 10B, C**), we observed a non-significant downregulation of fission adaptor protein *FIS1* across the obtained clones (ns; **Supplementary Figure S16C**). *MIEF1*, a known fission inhibitor, remained unchanged (**Supplementary Figure S16F**). In terms of mitochondrial fusion, we observed some differences in expression in heatmap (**Figure 10D**), with a non-significant decrease in the expression of *OMA1* and *NDUFAB1* in obtained clones (ns; **Supplementary Figures S16G, H**). However, there were no differences in the expression of key mitochondrial fusion regulators *TFAM*, *MFN1*, *MFN2*, and *OPA1* (**Supplementary Figures S16I–L**). Mitophagy-related genes were also assessed (**Figures 10E**, **S16M**), where *PINK1* showed no significant differences.

To evaluate the spatial distribution of mitochondria within the cells, we performed fluorescent microscopy using phalloidin to visualize the actin cytoskeleton and Mitotracker Orange to visualize mitochondria. There were no notable differences in mitochondrial localization in CLONE^2DG^ and CLONE^polyHEMA^ compared to ctrl (**Figures 10F**, **S17**-**19**).

Taken together, the lack of significant increase in either mitochondrial fusion or fission markers suggests that mitochondrial dynamics are not markedly altered in obtained clones.

To further explore the impact of adaptations of clones to selection on cellular energy metabolism, we analyzed key regulators and functional metabolism parameters. No significant changes in *HIF1A, NRF1* and *MTOR* expression were observed (**Supplementary Figure S20**). Interestingly, all three genes were downregulated in CLONE^ctrl^ compared to ctrl (ns).

**Figure 11:**
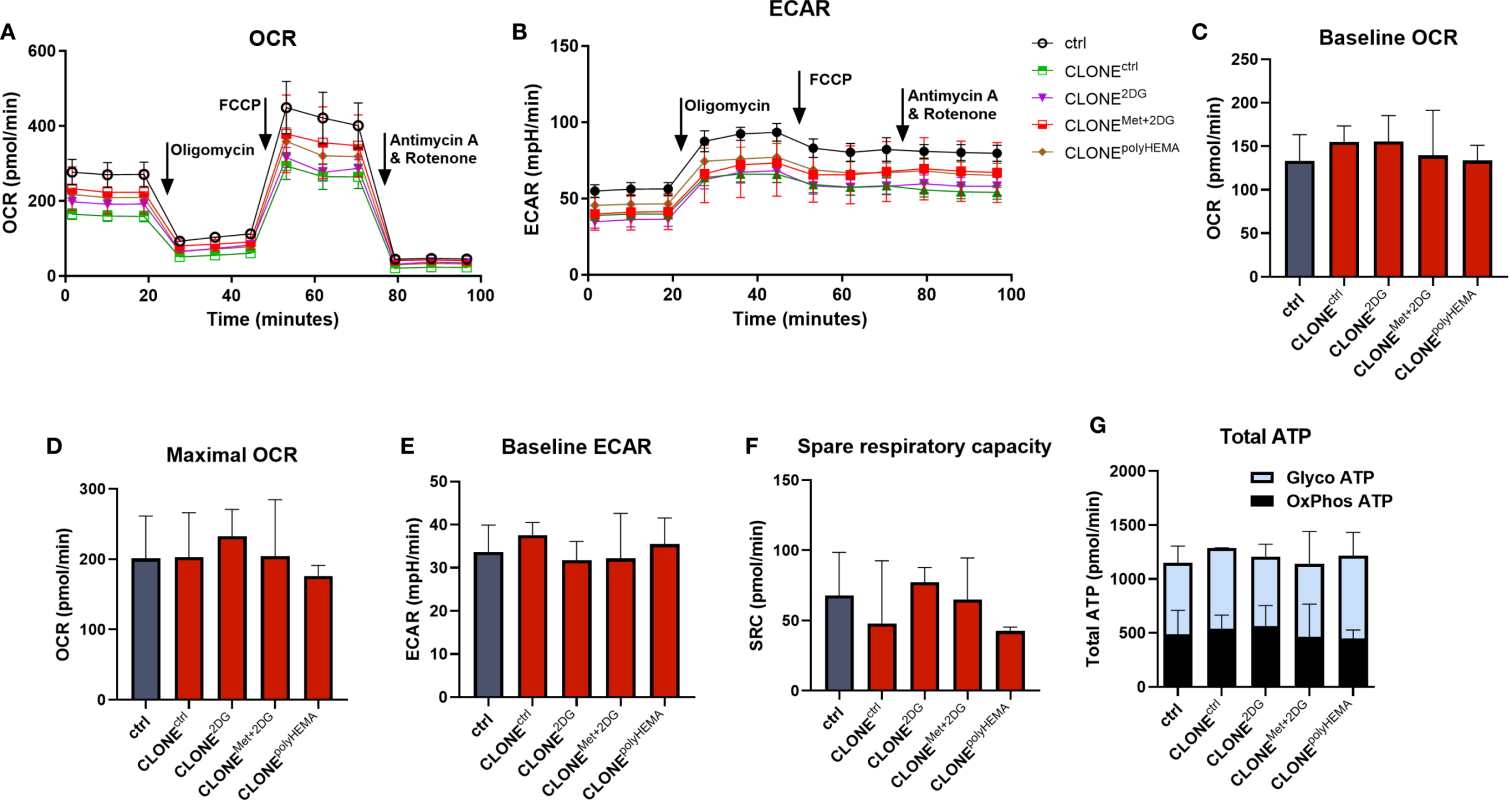
Cell energy homeostasis of adapted clones. MDA-MB-231 cells were grown in RPMI 1640 media with 1 g/L glucose and treated with 2-deoxy-D-glucose alone or in combination with metformin or grown on polyHEMA coated plates. After 72 hours, detached cells were reseeded in compound-free media for 72h. Reattached cells were reseeded again and treated with the same compounds. We have performed 4 cycles of detached cell selection. After the last cycle, detached cells were reseeded in compound free media and left to proliferate for 72 hours. Following selection, the baseline and maximal oxygen consumption rate (OCR) **(C, D)**, extracellular acidification rate (ECAR) **(E)** and spare respiratory capacity **(F)** were measured with Seahorse XFe24 analyzer using the Seahorse Real Time ATP Rate Assay. The results were normalized for relative cell number as determined by Hoechst staining. **(A, B)** Representative OCR and ECAR timelines are shown. **(G)** ATP production rates from glycolysis and oxidative phosphorylation were calculated using OCR following injections of oligomycin and rotenone plus antimycin A. Mean ± SEM is shown for three independent experiments. *p<0.05, **p<0.01, ***p<0.001, ****p<0.0001 versus attached control cells as determined by one-way ANOVA with Dunnett’s *post-hoc* test. Met, metformin; 2DG, 2-deoxy-D-glucose.

To evaluate mitochondrial respiration and glycolysis rate, we performed Seahorse XFe24 Mito stress test. Representative timelines for ECAR and OCR are shown in **Figures 11A, B**. No significant differences were detected among all clones in terms of baseline and maximal oxygen consumption rate (OCR), baseline extracellular acidification rate (ECAR) or spare respiratory capacity (SRC) (**Figures 11C–F**). In addition, there were also no differences in total ATP production, including GlycoATP (ATP produced by glycolysis) or OxPhosATP (ATP produced by oxidative phosphorylation) (**Figure 11G**). Overall, these results indicate that metabolic phenotype and mitochondrial function of the selected clones remained largely unchanged compared to the control, despite some differences at the transcriptional level.

It is important to note that during the treatment with 4.8 mM 2DG in 1 g/L glucose (5.6 mM), 2DG competitively inhibits glycolysis, reducing glycoATP by ~ 50% and also significantly affects N-glycosylation (51). In contrast, 0.6 mM 2DG (used in combination with 5 mM Met) is physiologically achievable, does not inhibit glycoATP, but affects protein N-glycosylation. 5 mM metformin at this concentration completely inhibits OCR. Altogether we can observe that in actively treated clones, the total ATP rate is not significantly altered as cells can compensate for the OCR inhibition with increased glycolysis for 5mM Met or *vice versa*, for 4.8mM 2DG, the OCR is upregulated (**Supplementary Figure S21**).

**Figure 12:**
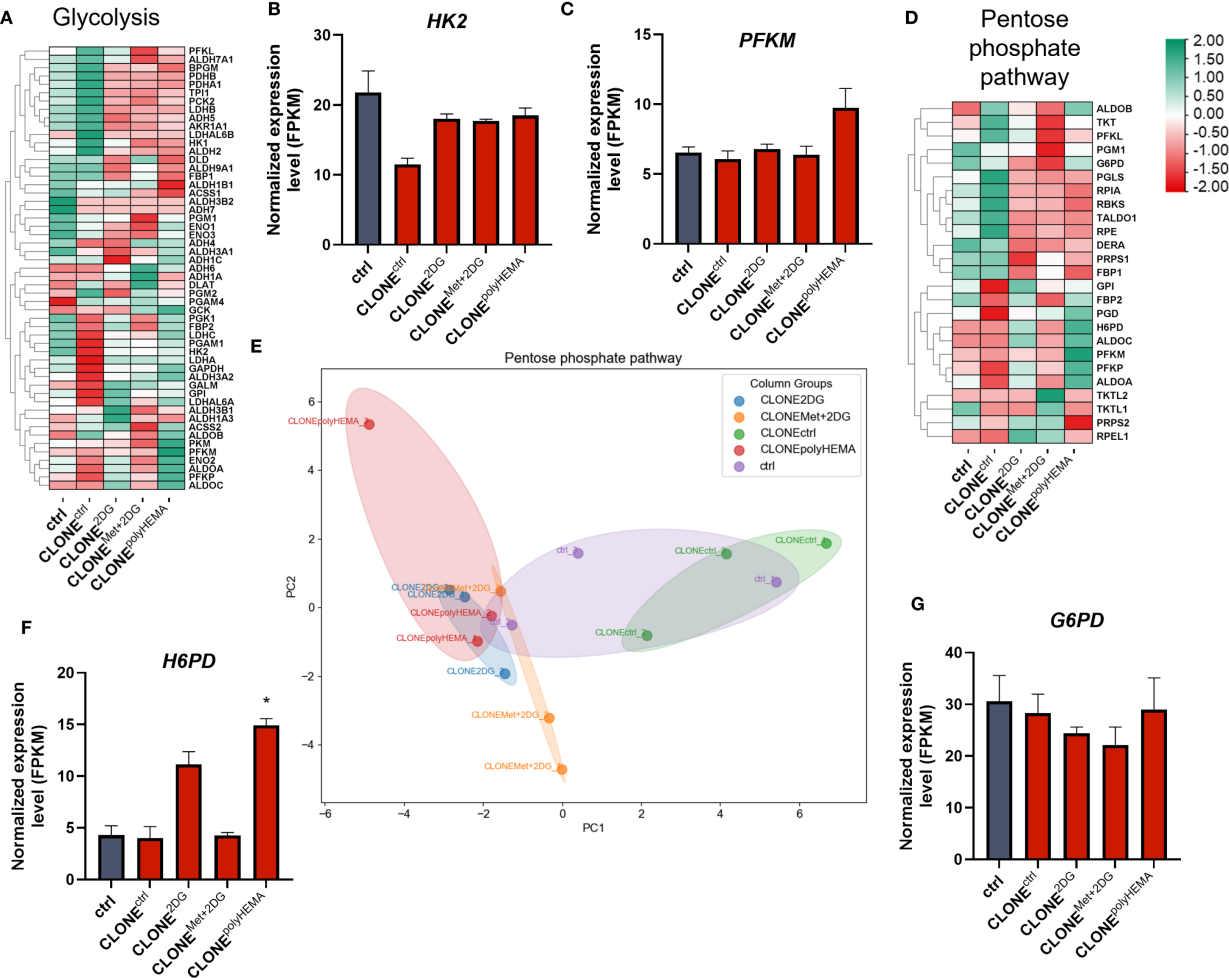
Transcriptomic analysis of glycolysis and pentose phosphate pathway. MDA-MB-231 cells were grown in RPMI 1640 media with 1 g/L glucose and treated with 2-deoxy-D-glucose alone or in combination with metformin or grown on polyHEMA coated plates. After 72 hours, detached cells were reseeded in compound-free media for 72h. Reattached cells were reseeded again and treated with the same compounds. We have performed 4 cycles of detached cell selection. After the last cycle, detached cells were reseeded in compound free media and left to proliferate for 72 hours. RNA expression levels of reattached cells were determined with RNA sequencing. **(A, D)** Heatmap of genes involved in glycolysis **(A)** and the pentose phosphate pathway **(D)**. Main genes in glycolysis **(B, C)** and pentose phosphate pathway **(F, G)** are shown in more detail. PCA analysis of pentose phosphate pathway **(E)**. The mean ± SEM for three independent experiments is shown. Normalized Z score values for three independent experiments are shown in heatmap. *p<0.05, **p<0.01, ***p<0.001, ****p<0.0001 versus attached control cells as determined by one-way ANOVA with Dunnett’s *post hoc* test. FPKM - fragments per kilobase of transcript per million mapped reads; Met, metformin; 2DG, 2-deoxy-D-glucose.

**Figure 13:**
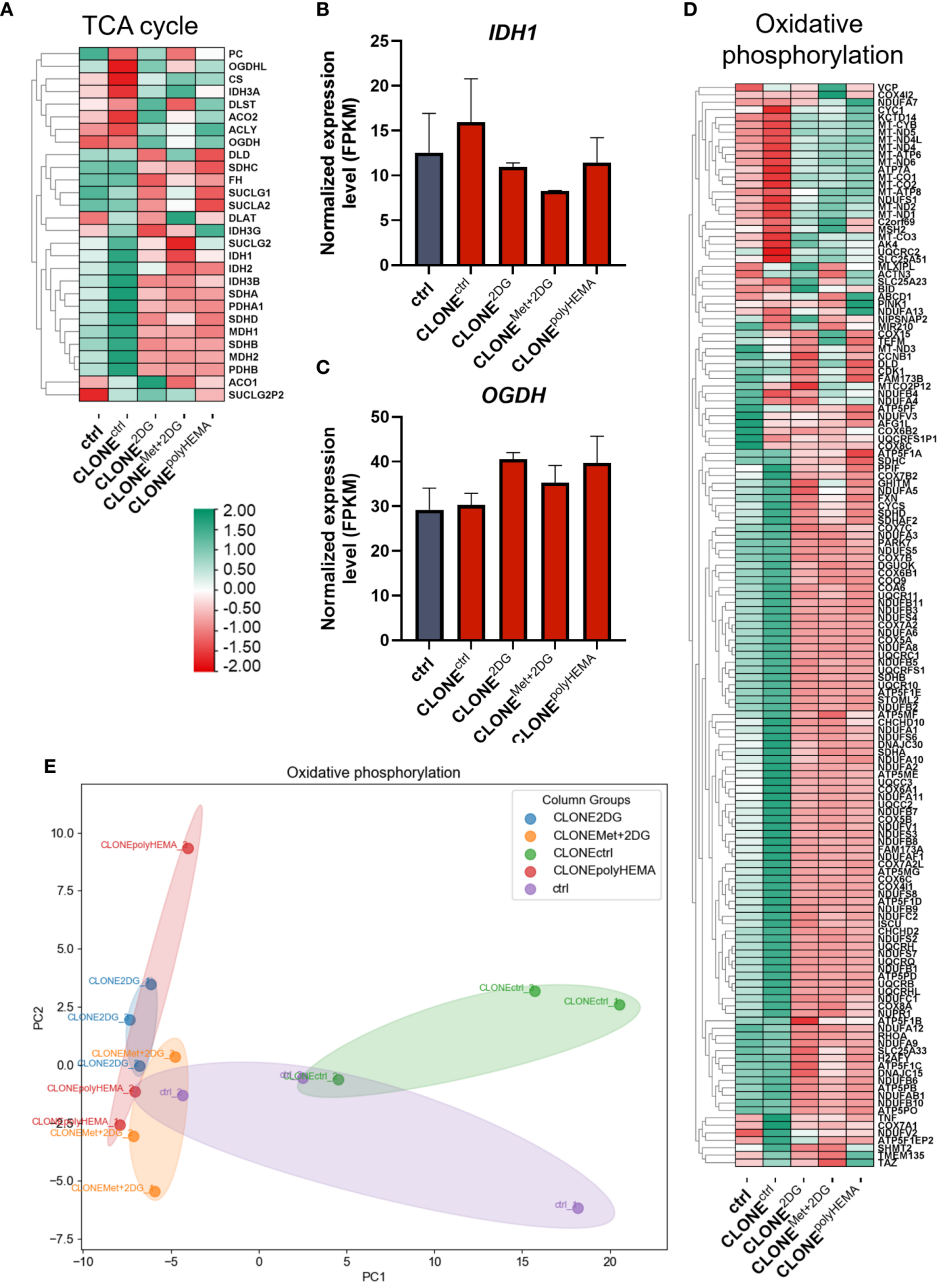
Transcriptomic analysis of TCA cycle and oxidative phosphorylation. MDA-MB-231 cells were grown in RPMI 1640 media with 1 g/L glucose and treated with 2-deoxy-D-glucose alone or in combination with metformin or grown on polyHEMA coated plates. After 72 hours, detached cells were reseeded in compound-free media for 72h. Reattached cells were reseeded again and treated with the same compounds. We have performed 4 cycles of detached cell selection. After the last cycle, detached cells were reseeded in compound free media and left to proliferate for 72 hours. RNA expression levels of reattached cells were determined with RNA sequencing. **(A, D)** Heatmap of genes involved in TCA cycle **(A)**, oxidative phosphorylation **(D)**. Main genes in TCA cycle **(B, C)** are shown in more detail. PCA analysis of oxidative phosphorylation **(E)**. The mean ± SEM for three independent experiments is shown. Normalized Z score values for three independent experiments are shown in heatmap. *p<0.05, **p<0.01, ***p<0.001, ****p<0.0001 versus attached control cells as determined by one-way ANOVA with Dunnett’s *post hoc* test. FPKM - fragments per kilobase of transcript per million mapped reads; Met, metformin; 2DG, 2-deoxy-D-glucose.

We next analyzed the transcriptional profile of genes involved in cellular metabolism. A general downregulation of glycolytic genes was observed in CLONE^2DG^, CLONE^Met+2DG^ and CLONE^polyHEMA^, compared to ctrl (**Figures 12A–C**, **S22**-**23**). *HK2* (ns) expression remained unchanged across all clones, while a modest increase in *PFKM* (ns) was detected only in CLONE^polyHEMA^ (**Figures 12B, C**). This is consistent with results from the Seahorse analysis, which showed no metabolic shift at the functional level. PCA analysis of glycolysis gene expression showed clustering of CLONE^2DG^, CLONE^Met+2DG^ and CLONE^polyHEMA^, while CLONE^ctrl^ clustered separately, and ctrl positioned in between (**Supplementary Figure S22**). Pentose phosphate pathway (PPP) also displayed downregulation of most pathway-related genes across the adapted clones (**Figures 12D–G**). Similarly, as for glycolysis, PCA analysis of PPP showed consistent clustering of CLONE^2DG^, CLONE^Met+2DG^ and CLONE^polyHEMA^, distinct from CLONE^ctrl^, with ctrl again positioned centrally (**Figure 12E**). Interestingly, *H6PD*, which catalyzes the first two reactions of PPP, was upregulated in CLONE^2DG^ (ns) and CLONE^polyHEMA^ (p<0.05) (**Figure 12F**). The key and rate limiting enzyme *G6PD* remained unchanged in all clones (**Figure 12G**). Transcriptomic analysis of the tricarboxylic acid (TCA) cycle revealed a general downregulation in CLONE^2DG^, CLONE^Met+2DG^ and CLONE^polyHEMA^, again with consistent clustering in PCA (**Figure 13A**, **S24**, **25**). Downregulation of TCA cycle was further confirmed with KEGG pathway analysis (**Supplementary Figure S25**). Among individual TCA genes, *IDH1* and *OGDH* expression remained unchanged (**Figures 13B-C**).

The adapted clones also showed downregulation of genes involved in oxidative phosphorylation, particularly those encoding subunits of ATP synthase (**Figures 13D**, **S26**). PCA analysis revealed that CLONE^2DG^, CLONE^Met+2DG^ and CLONE^polyHEMA^ clustered closely together, while CLONE^ctrl^ and ctrl formed a separate cluster on the opposite site (**Figure 13E**). Notably, the only OxPhos-related genes that were upregulated in the adapted clones were those encoded by mitochondrial DNA, suggesting a selective transcriptional response within the mitochondria. Fatty acid oxidation and metabolism were also differentially expressed (**Supplementary Figure S27**).

We next investigated the expression of genes involved in nucleotide metabolism, focusing on pyrimidine, purine, pyruvate, and one-carbon metabolism pathways. In pyrimidine metabolism, PCA analysis showed distinct clustering of CLONE^2DG^, CLONE^Met+2DG^ and CLONE^polyHEMA^, with CLONE^ctrl^ clustering oppositely, and ctrl in the middle, indicating clear transcriptional differences in this pathway despite no significant alterations in *CAD* or *DHODH* expression, two pyrimidine metabolism rate-limiting enzymes (**Supplementary Figure S28**). Purine metabolism seemed to be mostly downregulated in CLONE^2DG^, CLONE^Met+2DG^ and CLONE^polyHEMA^ compared to ctrl (**Supplementary Figure S29**). PCA analysis showed similar clustering as in pyrimidine metabolism. The main genes, *PPAT*, the first enzyme in this pathway, and *RRM2*, an enzyme involved in *de novo* biosynthesis of purine, showed a similar trend of upregulation only in CLONE^Met+2DG^ compared to ctrl (ns). Conversely, *ATIC* and *IMPDH1*, other important enzymes in this pathway, showed a decreasing trend (**Supplementary Figure S29**). KEGG pathways displayed consistent downregulation of genes involved in purine metabolism for CLONE^2DG^, CLONE^Met+2DG^ and CLONE^polyHEMA^, compared to ctrl (**Supplementary Figure S30**).

Analysis of pyruvate metabolism showed a downregulating trend in obtained clones (**Supplementary Figure S31**). Despite no differential expression of the first enzyme in the pathway, *PC*, *PDH1A*, and *PDHB* subunits of an enzyme that converts the energy from nutrients into acetyl-CoA, showed a decreasing trend in CLONE^2DG^, CLONE^Met+2DG^ and CLONE^polyHEMA^ (ns). PCA analysis showed similar results as in purine and pyrimidine metabolism (**Supplementary Figure S31**). Moreover, KEGG pathway analysis again displayed consistent downregulation of genes involved in pyruvate metabolism for CLONE^2DG^, CLONE^Met+2DG^ and CLONE^polyHEMA^, compared to ctrl (**Supplementary Figures S32**, **33**). Finally, one carbon metabolism was also differentially expressed in our obtained clones with no major differences for the main enzymes (*DHFR, SHMT, MTHFD1*) (**Supplementary Figure S34**).

## 4 Discussion

Cancer metastasis remains the leading cause of cancer-related mortality, including breast cancer. Triple negative breast cancer is especially aggressive and is known to metastasize in the distant organs such as the lung, liver and brain. Organ-specific dissemination has already been associated with distinct metastatic potential (81), where the tissue of origin influences both primary and metastatic tumor metabolism, potentially determining metastatic sites (82).

Some studies have shown that *in vitro* selection of the invasive MDA-MB-231 breast cancer cells that migrate through matrigel results in a stable, highly metastatic subpopulation capable of colonizing soft tissues, such as brain, ovary and lymph node, while the non-invasive clones tend to preferentially metastasize to bones (33, 83).

In this study, we investigated response of MDA-MB-231 triple negative breast cancer cells to repeated treatments that induce anchorage-independent growth. Selected clones were established through repeated cycles of detached cell selection following treatment with metabolic inhibitor 2DG (4.8 mM 2DG) or Met+2DG (5 mM metformin + 0.6 mM 2DG), or culturing on polyHEMA-coated plates, which prevent cell attachment.

Transcriptomic analysis revealed significant differential gene expression in the selected clones, with oxidative phosphorylation and chemical carcinogenesis through ROS as the most affected pathways in CLONE^2DG^, CLONE^Met+2DG^ and CLONE^polyHEMA^ compared to ctrl. Interestingly, OxPhos genes coded in mitochondrial DNA were upregulated in the selected clones, while OxPhos genes coded in cell nucleus were strongly downregulated (**Figure 13D**, **Supplementary Figure S26**). This indicates that while the machinery operates at a basal functional level, its capacity may be limited due to reduced transcription of structural components, particularly ATP synthase subunits. Downregulated OxPhos contrasts with prior studies that showed upregulation of OxPhos in anchorage-independent conditions (47, 77, 80, 84). In particular, enhanced mitochondrial oxidative phosphorylation has been demonstrated in TNBC metastasis using human patient-derived-xenograft models (85). Tumor mitochondrial functions are intimately linked to processes essential for tumor progression, including autophagy induction, anoikis resistance, EMT regulation, anchorage-independent growth and metastasis formation – often via increased ROS production and oncogene activation (19, 47, 77–79, 86). However, the discrepancy in obtained results could be attributed to the fact that the reattached, adapted clones, have returned to basal metabolic state post-selection and therefore no longer required increased OxPhos activity. Alternatively, the cells may have acquired anchorage-independence through alternative mechanisms. This hypothesis was supported by Seahorse Xe24 analysis, which showed no differences in either baseline or maximal OCR between adapted clones and ctrl (**Figure 11**). Despite the transcriptional regulation of nuclear OxPhos genes, ATP production remained unaffected in parallel with unaltered mitochondrial mass, suggesting post-transcriptional downregulation of compensation by mitochondrial-encoded components. This was further supported by low expression of master regulator of mitochondrial biogenesis *PPARGC1A* (**Supplementary Figure S16B**) and no differences in expression of *NRF1* (**Supplementary Figure S20C**), a key factor in mitochondrial DNA replication and EMT in breast cancer (87). This reinforces the notion that mitochondrial biogenesis was not induced in selected clones. In contrary to literature that links mitochondrial enlargement and fusion to survival in anchorage-independent conditions (47, 51, 77, 80, 88), we observed no differences in expression of mitochondrial fission regulators *OPA1* and *DNM1L*, and also no differences in fusion regulators. These results were supported by no differences in spare respiratory capacity. Given that mitochondrial fission supports rapid proliferation and redistribution of mitochondria to energy-demanding regions in the cell (89), its absence here is consistent with a senescent rather that proliferative phenotype in the adapted clones.

When focusing on glycolytic pathways, we observed no significant differences in glycoATP production or baseline ECAR between adapted clones and ctrl. These findings were further supported by transcriptomic analysis, which revealed no major alteration in the expression of key glycolytic genes (**Figures 12A-C**, **S22**, **23**). KEGG pathway analysis showed overall downregulation of glycolysis pathway, consistent with the mechanism of 2DG, which is a competitive glycolysis inhibitor. Altogether, these results indicate that the adapted clones retain a metabolic phenotype similar to that of the wild-type cells.

However, we observed an increased *H6PD* (p<0.05 in CLONE^polyHEMA^), a rate-limiting enzyme in PPP in CLONE^2DG^ and CLONE^polyHEMA^ compared to ctrl, while there were no differences in *G6PD* (**Figures 12D-G**). H6PD catalyzes the first two reactions of PPP by converting glucose-6-phosphate and NADP(+), indicating increased metabolite flow through this pathway, however, most of PPP genes were downregulated. PPP is crucial in cancer metastasis and progression as it provides ribose-5-phosphate for nucleotide biosynthesis and generates NADPH, essential for macromolecule synthesis and ROS scavenging (23, 90). Upregulation of PPP has previously been identified as a key feature of anchorage-independent growth and remained upregulated in detached cancer cells (47, 70, 77), in contrary to our observations.

ROS play a significant role in cancer progression and many different mechanisms can lead to increased ROS biosynthesis, such as PPP activation, mitochondrial activity, cell detachment and ER stress (47, 91–93). In this study, we observed increased cellular ROS levels in both attached and detached treated clones, compared to treated wild-type cells and ctrl (**Figure 6A**). Notably, following reattachment, ROS levels remained modestly elevated (up to 120% of the ctrl) in obtained clones (**Figure 6B**). Interestingly, the expression of key scavenging enzymes, including *GPX1* and *SOD1*, was decreased. Low ROS levels, such as those generated during OxPhos can also act as signaling molecules that promote EMT, further emphasizing the potential role of ROS in the observed phenotypes.

Increased metabolites from the TCA cycle are essential for fueling OxPhos, which is especially important in metastatic cells (77, 85). Contrary to this, we observed downregulated TCA cycle in the adapted clones, with no differences in *IDH1* expression (**Figures 13A–C**). Interestingly, while pyrimidine metabolism remained unchanged, purine metabolism was downregulated in the adapted clones. *De novo* nucleotide synthesis is crucial for rapidly proliferating cells and has been proposed as a metabolic hallmark of metastatic breast cancer cells (94). In agreement with no increase in the total cell number after re-attachment of the selected cells (CLONE cells), this suggests that cells must first overcome the stress of detachment, before they can resume proliferation.

Nutrient availability plays a crucial role in dictating metabolic behavior (69), particularly within the tumor microenvironment, where glucose levels, oxygen availability and pH values are typically decreased (45, 95). For instance, acidic environment has been shown to enhance cancer cell detachment (45, 96). A recent study demonstrated that pancreatic cancer clones adapted to low glucose and glutamine conditions exhibited increased proliferation *in vitro* and enhanced tumor-forming capacity *in vivo* (97). Repeated treatment with glycolysis inhibition with 2DG can partially mimic glucose-deprived conditions of tumor microenvironment. Interestingly, the glycoATP production rate in the adapted clones remained unchanged compared to ctrl. It would be worthwhile to analyze whether these adapted clones also display enhanced tumor-forming potential *in vivo* under such treatment conditions.

Energy homeostasis and redox balance are often restored through oncogene activation (23–28). In addition to their metabolic functions, mitochondria can increase ROS production, which, through activation of oncogenic signaling pathways promotes cancer metastasis (19, 47, 77–79, 86). In our adapted clones, the proto-oncogene *MYC* was upregulated, correlating with enhanced cell survival. Alongside downregulated apoptotic signaling, we also observed upregulation of other stemness markers, consistent with observed phenotypic changes (**Figures 9**, **S13**).

Another cancer hallmark is deregulated cell cycle (98–100). For tumor expansion and metastasis, cancer cells must sustain continuous growth and division, while circulating tumor cells typically display reduced proliferation and stemness. In our model of adapted clones, we observed no statistically significant upregulation of cell cycle progression genes (**Figures 8**, **S12**). We have observed a small upregulation of *CDK6* (ns), while *CDK4* (ns) showed a decreasing trend in selected clones. Inhibition of CDK4/6 has been reported to trigger senescence, apoptosis or quiescence in certain cancers (101–103). However, since we did not observe increased cell death in reattached adapted clones, we speculate that downregulation of *CDK4* does not play a major role in inducing apoptosis in this context. *CCND1*, cyclin D1, is a key regulator of cell cycle progression and oncogenic driver in various cancers (104–106), including breast cancer (107), where it is overexpressed in up to 50% of cases and is associated with poor prognosis (108). Despite its upregulation (ns) in our adapted clones, we have not observed increased proliferation. Cyclin D1 is also known to inhibit PPARα, a key metabolic transcription factor, that promotes fatty acid oxidation (109), linking cell cycle control with altered metabolic states. Additionally, a trend of downregulated *RPS6* (ns) may indicate a cell cycle arrest at G0-G1 phase, rather than apoptosis (110), aligning with the lack of increased cell death after reattachment. Transcriptomic analysis suggests that the cells enter a senescent-like state (**Supplementary Figures S14, S15**) with absent mitochondrial fission (89) and downregulated apoptotic pathways. The concurrent upregulation of stemness markers further reinforces the idea of a stress-adapted, survival-prone cellular phenotype in the selected CLONES. Moreover, cell cycle is typically inhibited in the detached state, successful proliferation post reattachment would require reactivation of the cycle to overcome detachment-induced stress.

Glycosylation has been shown to play a crucial role in cancer metastasis (70, 111–114) and our prior work also demonstrated that N-glycosylation inhibition can induce cancer cell detachment, elicit ER stress and activate UPR (70). Interestingly, the re-attached clones exhibited a similar UPR gene expression profile as one-time treated cells (**Figures 5**, **S5**), indicating that detachment imposes significant cellular stress, and extended recovery time may be required. Additionally, genes involved in N-glycan biosynthesis, N-glycan branching and OST complex, showed complete opposite gene expression compared to ctrl (**Figures 5**, **S6**). These findings suggest sustained inhibition of N-glycosylation, potentially as a result of prolonged cell detachment, continuous treatment with N-glycosylation inhibitors, or a combination of both.

Notably, UPR activation has been shown to be upregulated in metastatic breast cancer cells, supporting our findings (70). PCA analysis showed similar clustering of clones in UPR and regulation of ROS metabolism showing similar response in both pathways. In the current study, we observed no significant alterations in ER stress pathway with slight increase in *ATF6* expression, accompanied by very low expression of *BCL2*. This suggests that cells are undergoing ER stress recovery rather than apoptosis.

To investigate whether our adapted clones exhibit a mesenchymal phenotype, we analyzed the expression of EMT markers (44, 115) (**Figures 4**, **S4**). We have observed upregulation of several mesenchymal markers, *SNAI1*, *SNAI2* and *FN1* (ns for all). In contrast, epithelial markers showed variable expression in selected clones. Interestingly, metformin has been shown to reduce the expression of EMT markers at the metastases site (116), which may account for the differences observed in CLONE^2DG^ and CLONE^Met+2DG^. Moreover, *SNAI1* is known to promote the expansion of a stem cell–like population that is resistant to apoptosis and capable of self-renewal (117), aligning with our observation of increased stemness in adapted clones. Overall, our results suggest that the adapted clones have acquired mesenchymal traits, facilitating anchorage-independent survival. However, the concurrent upregulation of certain epithelial markers may enable reattachment and proliferation at secondary sites. Oxidative stress and moderate increase in ROS levels, as observed in our adapted clones, can activate the pro-tumorigenic and pro-inflammatory transcription factor NF-κB (91–93). To explore this pathway further, we analyzed key components in NF-κB regulation (**Figures 7A-B**, **S9**). Similar to what we previously observed in detached cells (70) and consistent with the modest increase in ROS levels, *NFKB2* was upregulated (p<0.05 in CLONE^ctrl^ and CLONE^2DG^). In contrast, the deubiquitinase *CSN5* was upregulated in CLONE^ctrl^ (p<0.05), suggesting increased NF‐κB stability and protection from ubiquitin-mediated degradation. Interestingly, the expression pattern closely mirrors what we previously observed in MDA-MB-231 cells following detachment after single treatment, with either 4.8 2DG or Met+0.6 2DG. These findings suggest that NF-κB activation in adapted clones is sustained and likely contributes to their survival and inflammatory signaling under anchorage-independent conditions.

We next analyzed the impact of cell reattachment following consecutive treatment with the N-glycosylation inhibitor 2DG on PD-L1 expression. PD-L1 has been shown to be expressed in 20% TNBC tumors, suggesting that antibodies targeting PD-1 or PD-L1 may have utility as a novel therapeutic strategy in TNBC (118). Interestingly, one study suggested using PD-L1 status as predictive marker of response to standard neoadjuvant therapy and that the PD-L1-positivity might serve as a factor supporting the clinician’s decision about less aggressive therapy (119). Consistent with earlier findings (51, 70), we observed decreased surface PD-L1 expression in the treated clones, as well as after reattachment in compound-free media. Thus, decreased PD-L1 suggests that therapies with antibodies against PD-1 or CTLA-4 could be more beneficial than targeting PD-L1. Earlier findings have also shown the connection between PD-L1 expression and AMPK activation (51, 70). However, despite decreased surface PD-L1 levels, *CD274* expression was upregulated (ns) (**Figures 7C-F**). This apparent discrepancy is in line with previous studies showing that PD-L1 expression is regulated at multiple levels, including gene amplification, chromatin remodeling, transcription, post-transcriptional regulation, translation and post-translational modifications (118, 120). Although NF-κB has been shown to enhance PD-L1 expression in various cancers (121–123), we have previously shown that impaired protein N-glycosylation exerts a stronger influence on downregulation of surface PD-L1 expression, than NF-κB-mediated gene stabilization (51, 70). This highlights the importance of post-transcriptional and post-translational mechanisms, such as reduced N-glycosylation and ER stress, in regulating PD-L1 surface expression. Additionally, the metalloproteases, ADAM10 and ADAM17, are known to mediate PD-L1 cleavage in breast cancer cells, leading to the release of soluble PD-L1 into the extracellular space (76). In our clones, both metalloproteases, especially *ADAM17* showed a trend of upregulation (ns), which may further explain the observed reduction in surface PD-L1 levels despite upregulated *CD274* transcription.

In summary, we have analyzed selected clones of MDA-MB-231 breast cancer cells that model metastatic behavior following re-attachment at secondary location. These adapted clones demonstrated increased detachment capacity, upregulated proto-oncogenes, and inhibited cell cycle progression. Clones generated through selection of detached cells induced by N-glycosylation inhibitors or growth on polyHEMA-coated plates, exhibited similar phenotypes, distinct from untreated control. Notably, cells derived from spontaneous detachment (without added treatment), CLONE^ctrl^, more closely resembled control but displayed more pronounced gene expression with changes in different pathways, such as EMT, cell cycle, apoptosis and UPR. CLONE^2DG^, CLONE^Met+2DG^ and CLONE^polyHEMA^ exhibited strong downregulation of OxPhos-related transcripts yet maintained OxPhos ATP production. UPR was also upregulated, highlighting their role in anoikis resistance and adaptation to detachment-induced stress. The lack of upregulation of mitochondrial fission suggests cell senescence rather than fast proliferation. Collectively, our data suggests that reattached cells are well adapted to anchorage-independent growth, primed to survive in a new environment and currently express senescent-like phenotype. Despite all these adaptations, these cells have decreased PD-L1 expression, which makes them more vulnerable and an easier target for immune evasion.

## 5 Conclusions

Metastasis, the dissemination of cancer cells to distant sites, represents the primary cause of mortality in cancer patients. However, the complex steps involved in this process remain poorly understood due to difficulties in *in vivo* observation. While *in vitro* models effectively capture early events like tumor budding (35, 36), the critical reattachment phase of disseminated cancer cells is frequently overlooked.

To address this, we generated novel clones from the triple-negative breast cancer (TNBC) MDA-MB-231 cell line that mimics metastatic behavior, specifically their enhanced capacity for re-attachment and survival. These adapted clones were derived through serial selection of detached cell populations, using either specific metabolic treatments 2DG alone or combined with metformin or culture on polyHEMA-coated surfaces. Characterization revealed that these clones exhibited upregulated epithelial-to-mesenchymal transition (EMT) markers, signifying increased detachment propensity, alongside an upregulation of mesenchymal markers, likely facilitating re-adhesion and proliferation.

A striking finding was the consistent upregulation of both stemness and senescence markers across all clones, consistent with no increase in cell cycle progression genes. This, coupled with unchanged pyrimidine, purine metabolism and mitochondrial fission, suggests that these cells adopt a senescent, dormant-like state, a key adaptation for survival in the metastatic niche. Furthermore, our results indicated that N-glycosylation inhibitors or prolonged detachment induced significant endoplasmic reticulum (ER) stress and activated unfolded protein response (UPR) pathways in the reattached clones. Despite these cellular adaptations, the overall energy metabolism of these clones, as measured by glycoATP and OxPhos-derived ATP production, remained comparable to untreated control cells, aligning with previous metabolomic data. Despite upregulated *CD274* (PD-L1) transcription, potentially mediated by pNF-κB gene stabilization, surface PD-L1 expression consistently remained decreased in both detached and reattached treated clones. This suggests a post-transcriptional regulatory mechanism, possibly involving ER stress, inhibition of protein N-glycosylation, or ADAM protease-mediated cleavage.

In summary, our adapted clones model crucial aspects of early metastatic behavior, demonstrating enhanced detachment, upregulation of proto-oncogenes, and a senescent-like phenotype characterized by inhibited cell cycle progression, downregulated nuclear-encoded OXPHOS transcripts (despite preserved ATP production), and elevated ER stress and UPR signaling. While remarkably adapted to anchorage-independent survival, the observed reduction in surface PD-L1 expression in these clones potentially renders them more vulnerable to immune surveillance, suggesting novel therapeutic avenues.

## Data Availability

The datasets presented in this study can be found in online repositories. The names of the repository/repositories and accession number(s) can be found below: https://www.ncbi.nlm.nih.gov/, https://www.ncbi.nlm.nih.gov/geo/query/acc.cgi?acc=GSE279580.
