# Supplementary material for: MDA-MB-231 breast cancer cells adapted to anchorage-independent growth reveal senescent-like phenotype and persistent downregulation of PD-L1 expression
Source: Front Oncol. 2025 Oct 1;15:1667308. doi: 10.3389/fonc.2025.1667308 (PMC12520971; doi:10.3389/fonc.2025.1667308)
Supplement: Supplementary file 1 [file DataSheet1.docx]

Supplementary Material

**Figure S1 Percentage detached cells in the live population of treated adapted clones.** MDA-MB-231 cells were grown in RPMI 1640 media with 1 g/L glucose and treated with 2-deoxy-D-glucose alone or in combination with metformin. After 72 hours, detached cells were reseeded in compound-free media for 72h. Reattached cells were reseeded again and treated with the same compounds. We have performed 4 cycles of detached cell selection. After the last cycle, detached and attached cells were collected separately, stained with trypan blue and counted with cell counter Countess. % detached cells in the live population was determined. The mean ± SEM for three independent experiments is shown. *p<0.05, **p<0.01, ***p<0.001, ****p<0.0001 versus attached control cells as determined by one-way ANOVA with Dunnett’s post hoc test. Met – metformin; 2DG - 2-deoxy-D-glucose.

**Figure S2 KEGG analysis of differential gene expression**. MDA-MB-231 cells were grown in RPMI 1640 media with 1 g/L glucose and treated with 2-deoxy-D-glucose alone or in combination with metformin. After 72 hours, detached cells were reseeded in compound-free media for 72h. Reattached cells were reseeded again and treated with the same compounds. We have performed 4 cycles of detached cell selection. After the last cycle, detached cells were reseeded in compound free media and left to proliferate for 72 hours. RNA expression levels of reattached cells were determined with RNA sequencing. KEGG analysis of differentially expressed genes in CLONE^ctrl^ (A), CLONE^2DG^ (B), CLONE^Met+2DG^ (C) or CLONE^polyHEMA^ (D) cells versus ctrl, represented ten most significantly differentially expressed pathways. Met – metformin; 2DG - 2-deoxy-D-glucose.

**Figure S3 Gene ontology analysis of differential gene expression.** MDA-MB-231 cells were grown in RPMI 1640 media with 1 g/L glucose and treated with 2-deoxy-D-glucose alone or in combination with metformin. After 72 hours, detached cells were reseeded in compound-free media for 72h. Reattached cells were reseeded again and treated with the same compounds. We have performed 4 cycles of detached cell selection. After the last cycle, detached cells were reseeded in compound free media and left to proliferate for 72 hours. RNA expression levels of reattached cells were determined with RNA sequencing. Gene ontology analysis of differentially expressed genes in CLONE^ctrl^ (A), CLONE^2DG^ (B), CLONE^Met+2DG^ (C) or CLONE^polyHEMA^ (D) cells versus ctrl, represented ten most significantly differentially expressed pathways. MF – molecular function; CC – cellular component; BP – biological process; Met – metformin; 2DG - 2-deoxy-D-glucose.

**Figure S4 Transcriptomic analysis of genes involved in EMT.** MDA-MB-231 cells were grown in RPMI 1640 media with 1 g/L glucose and treated with 2-deoxy-D-glucose alone or in combination with metformin. After 72 hours, detached cells were reseeded in compound-free media for 72h. Reattached cells were reseeded again and treated with the same compounds. We have performed 4 cycles of detached cell selection. After the last cycle, detached cells were reseeded in compound free media and left to proliferate for 72 hours. RNA expression levels of reattached cells were determined with RNA sequencing. Heatmap (A) of EMT. Main genes in EMT are shown in more detail (B-C). Normalized Z score values for three independent experiments are shown in heatmap. The mean ± SEM for three independent experiments is shown. *p<0.05, **p<0.01, ***p<0.001, ****p<0.0001 versus attached control cells as determined by one-way ANOVA with Dunnett’s post hoc test. FPKM - fragments per kilobase of transcript per million mapped reads. Met – metformin; 2DG - 2-deoxy-D-glucose.

**Figure S5** **Transcriptomic analysis of genes involved in UPR.** MDA-MB-231 cells were grown in RPMI 1640 media with 1 g/L glucose and treated with 2-deoxy-D-glucose alone or in combination with metformin. After 72 hours, detached cells were reseeded in compound-free media for 72h. Reattached cells were reseeded again and treated with the same compounds. We have performed 4 cycles of detached cell selection. After the last cycle, detached cells were reseeded in compound free media and left to proliferate for 72 hours. RNA expression levels of reattached cells were determined with RNA sequencing. Heatmap (A) and principal component analysis (B) of unfolded protein response. Main genes in UPR and ER stress are shown in more detail (C-L). Normalized Z score values for three independent experiments are shown in heatmap. The mean ± SEM for three independent experiments is shown. *p<0.05, **p<0.01, ***p<0.001, ****p<0.0001 versus attached control cells as determined by one-way ANOVA with Dunnett’s post hoc test. FPKM - fragments per kilobase of transcript per million mapped reads. Met – metformin; 2DG - 2-deoxy-D-glucose.

**Figure S6 Transcriptomic analysis of genes involved in oligosaccharyltransferase.** MDA-MB-231 cells were grown in RPMI 1640 media with 1 g/L glucose and treated with 2-deoxy-D-glucose alone or in combination with metformin. After 72 hours, detached cells were reseeded in compound-free media for 72h. Reattached cells were reseeded again and treated with the same compounds. We have performed 4 cycles of detached cell selection. After the last cycle, detached cells were reseeded in compound free media and left to proliferate for 72 hours. RNA expression levels of reattached cells were determined with RNA sequencing. Heatmap of oligosaccharyltransferase (A). *DPAGT1* is shown in detail (B). Normalized Z score values for three independent experiments are shown in heatmap. The mean ± SEM for three independent experiments is shown. *p<0.05, **p<0.01, ***p<0.001, ****p<0.0001 versus attached control cells as determined by one-way ANOVA with Dunnett’s post hoc test. FPKM - fragments per kilobase of transcript per million mapped reads. Met – metformin; 2DG - 2-deoxy-D-glucose.

**Figure S7 KEGG pathways analysis of N-glycan biosynthesis.** MDA-MB-231 cells were grown in RPMI 1640 media with 1 g/L glucose and treated with 2-deoxy-D-glucose alone or in combination with metformin. After 72 hours, detached cells were reseeded in compound-free media for 72h. Reattached cells were reseeded again and treated with the same compounds. We have performed 4 cycles of detached cell selection. After the last cycle, detached cells were reseeded in compound free media and left to proliferate for 72 hours. RNA expression levels of reattached cells were determined with RNA sequencing. KEGG pathway analysis for N-glycan biosynthesis is shown. The mean for three independent experiments is shown. Met – metformin; 2DG - 2-deoxy-D-glucose.

**Figure S8** **Transcriptomic analysis of genes involved in ROS.** MDA-MB-231 cells were grown in RPMI 1640 media with 1 g/L glucose and treated with 2-deoxy-D-glucose alone or in combination with metformin. After 72 hours, detached cells were reseeded in compound-free media for 72h. Reattached cells were reseeded again and treated with the same compounds. We have performed 4 cycles of detached cell selection. After the last cycle, detached cells were reseeded in compound free media and left to proliferate for 72 hours. RNA expression levels of reattached cells were determined with RNA sequencing. Main genes in ROS scavenging are shown in more detail. The mean ± SEM for three independent experiments is shown. *p<0.05, **p<0.01, ***p<0.001, ****p<0.0001 versus attached control cells as determined by one-way ANOVA with Dunnett’s post hoc test. FPKM - fragments per kilobase of transcript per million mapped reads. Met – metformin; 2DG - 2-deoxy-D-glucose.

**Figure S9 Transcriptomic analysis of genes involved in canonical NF-κB pathway.** MDA-MB-231 cells were grown in RPMI 1640 media with 1 g/L glucose and treated with 2-deoxy-D-glucose alone or in combination with metformin. After 72 hours, detached cells were reseeded in compound-free media for 72h. Reattached cells were reseeded again and treated with the same compounds. We have performed 4 cycles of detached cell selection. After the last cycle, detached cells were reseeded in compound free media and left to proliferate for 72 hours. RNA expression levels of reattached cells were determined with RNA sequencing. Heatmap for canonical NF-κB pathway (A). *STUB1* (B) and *CSN5* (C) gene expressions are shown in more detail. The mean ± SEM for three independent experiments is shown. Normalized Z score values for three independent experiments are shown in heatmap. *p<0.05, **p<0.01, ***p<0.001, ****p<0.0001 versus attached control cells as determined by one-way ANOVA with Dunnett’s post hoc test. FPKM - fragments per kilobase of transcript per million mapped reads. Met – metformin; 2DG - 2-deoxy-D-glucose.

**Figure S10** **Transcriptomic analysis of genes involved in PD-L1 pathway.** MDA-MB-231 cells were grown in RPMI 1640 media with 1 g/L glucose and treated with 2-deoxy-D-glucose alone or in combination with metformin or grown on polyHEMA coated plates. After 72 hours, detached cells were reseeded in compound-free media for 72h. Reattached cells were reseeded again and treated with the same compounds. We have performed 4 cycles of detached cell selection. After the last treatment, detached and attached cells were collected separately and surface PD-L1 (A) was determined with flow cytometry, where the mean fluorescence intensity is shown. After the last cycle, detached cells were also reseeded in compound free media and left to proliferate for 72 hours, and the re-attached cells were also collected where surface PD-L1 was again analyzed with flow cytometry (B). Gating strategy for PD-L1 gating is shown in (C). RNA expression levels of reattached cells were determined with RNA sequencing. *ADAM10* (D) and *ADAM17* (E) gene expressions are shown in more detail. The mean ± SEM for three independent experiments is shown. *p<0.05, **p<0.01, ***p<0.001, ****p<0.0001 versus attached control cells as determined by one-way ANOVA with Dunnett’s post hoc test. FPKM - fragments per kilobase of transcript per million mapped reads; MFI – mean fluorescence intensity; RFU – relative fluorescence unit; Met – metformin; 2DG - 2-deoxy-D-glucose.

**Figure S11** **KEGG pathways analysis of PD-L1 expression.** MDA-MB-231 cells were grown in RPMI 1640 media with 1 g/L glucose and treated with 2-deoxy-D-glucose alone or in combination with metformin. After 72 hours, detached cells were reseeded in compound-free media for 72h. Reattached cells were reseeded again and treated with the same compounds. We have performed 4 cycles of detached cell selection. After the last cycle, detached cells were reseeded in compound free media and left to proliferate for 72 hours. RNA expression levels of reattached cells were determined with RNA sequencing. KEGG pathway analysis for PD-L1 expression is shown. The mean for three independent experiments is shown. Met – metformin; 2DG - 2-deoxy-D-glucose.

**Figure S12** **Transcriptomic analysis of cell cycle.** MDA-MB-231 cells were grown in RPMI 1640 media with 1 g/L glucose and treated with 2-deoxy-D-glucose alone or in combination with metformin. After 72 hours, detached cells were reseeded in compound-free media for 72h. Reattached cells were reseeded again and treated with the same compounds. We have performed 4 cycles of detached cell selection. After the last cycle, detached cells were reseeded in compound free media and left to proliferate for 72 hours. RNA expression levels of reattached cells were determined with RNA sequencing. Cell cycle gene expression is shown in more detail (A-F). The mean ± SEM for three independent experiments is shown. *p<0.05, **p<0.01, ***p<0.001, ****p<0.0001 versus attached control cells as determined by one-way ANOVA with Dunnett’s post hoc test. FPKM - fragments per kilobase of transcript per million mapped reads. Met – metformin; 2DG - 2-deoxy-D-glucose.

**Figure S13** **Transcriptomic analysis of cell stemness.** MDA-MB-231 cells were grown in RPMI 1640 media with 1 g/L glucose and treated with 2-deoxy-D-glucose alone or in combination with metformin. After 72 hours, detached cells were reseeded in compound-free media for 72h. Reattached cells were reseeded again and treated with the same compounds. We have performed 4 cycles of detached cell selection. After the last cycle, detached cells were reseeded in compound free media and left to proliferate for 72 hours. RNA expression levels of reattached cells were determined with RNA sequencing. Cell stemness gene expression is shown in more detail. The mean ± SEM for three independent experiments is shown. *p<0.05, **p<0.01, ***p<0.001, ****p<0.0001 versus attached control cells as determined by one-way ANOVA with Dunnett’s post hoc test. FPKM - fragments per kilobase of transcript per million mapped reads. Met – metformin; 2DG - 2-deoxy-D-glucose.

**Figure S14** **Analysis of genes involved in senescence.** MDA-MB-231 cells were grown in RPMI 1640 media with 1 g/L glucose and treated with 2-deoxy-D-glucose alone or in combination with metformin. After 72 hours, detached cells were reseeded in compound-free media for 72h. Reattached cells were reseeded again and treated with the same compounds. We have performed 4 cycles of detached cell selection. After the last cycle, detached cells were reseeded in compound free media and left to proliferate for 72 hours. RNA expression levels of reattached cells were determined with RNA sequencing. (A) Heatmap of expression of genes involved in cell senescence. (B) PCA analysis of genes involved in senescence. Normalized Z score values for three independent experiments are shown in heatmap. Met – metformin; 2DG - 2-deoxy-D-glucose.

Figure S15 KEGG pathway analysis of cellular senescence. MDA-MB-231 cells were grown in RPMI 1640 media with 1 g/L glucose and treated with 2-deoxy-D-glucose alone or in combination with metformin. After 72 hours, detached cells were reseeded in compound-free media for 72h. Reattached cells were reseeded again and treated with the same compounds. We have performed 4 cycles of detached cell selection. After the last cycle, detached cells were reseeded in compound free media and left to proliferate for 72 hours. RNA expression levels of reattached cells were determined with RNA sequencing. KEGG pathway analysis for cellular senescence is shown. The mean for three independent experiments is shown. Met – metformin; 2DG - 2-deoxy-D-glucose.

**Figure S16 Analysis of mitochondrial dynamics in adapted clones.** MDA-MB-231 cells were grown in RPMI 1640 media with 1 g/L glucose and treated with 2-deoxy-D-glucose alone or in combination with metformin. After 72 hours, detached cells were reseeded in compound-free media for 72h. Reattached cells were reseeded again and treated with the same compounds. We have performed 4 cycles of detached cell selection. After the last cycle, attached and detached cells were collected separately and relative mitochondrial mass through mitotracker orange (A) was determined with flow cytometry. After the last cycle, detached cells were reseeded in compound free media and left to proliferate for 72 hours. RNA expression levels of reattached cells were determined with RNA sequencing. Gene expression of mitochondrial dynamics is shown in more detail (B-N). The mean ± SEM for three independent experiments is shown. *p<0.05, **p<0.01, ***p<0.001, ****p<0.0001 versus attached control cells as determined by one-way ANOVA with Dunnett’s post hoc test. FPKM - fragments per kilobase of transcript per million mapped reads. Met – metformin; 2DG - 2-deoxy-D-glucose.

**Figure S17 Localization of mitochondria in control MDA-MB-231 cells.** MDA-MB-231 cells were grown in RPMI 1640 media with 1 g/L glucose for eight passages. After the last reseeding, cells were seeded on coverslips for 24 hours. Phalloidin and mitotracker orange fluorescence were detected with fluorescent microscopy. The bar represents 32 µm.

**Figure S18** **Localization of mitochondria in CLONE^2DG^ cells.** MDA-MB-231 cells were grown in RPMI 1640 media with 1 g/L glucose and treated with 4.8 mM 2-deoxy-D-glucose. After 72 hours, detached cells were reseeded in compound-free media for 72h. Reattached cells were reseeded again and treated with the same compound. We have performed 4 cycles of detached cell selection. After the last cycle, detached cells were reseeded in compound free media and left to proliferate for 72 hours. After 72 hours, cells were seeded on coverslips for 24 hours. Phalloidin and mitotracker orange fluorescence were detected with fluorescent microscopy. The bar represents 32 µm.

**Figure S19** **Localization of mitochondria in CLONE^polyHEMA^ cells.** MDA-MB-231 cells were grown in RPMI 1640 media with 1 g/L glucose and reseeded on polyHEMA-coated plates. After 72 hours, detached cells were reseeded in compound-free media for 72h. Reattached cells were reseeded again on polyHEMA-coated plates. We have performed 4 cycles of detached cell selection. After the last cycle, detached cells were reseeded in compound free media and left to proliferate for 72 hours. After 72 hours, cells were seeded on coverslips for 24 hours. Phalloidin and mitotracker orange fluorescence were detected with fluorescent microscopy. The bar represents 32 µm.

**Figure S20** **Transcriptomic analysis of *HIF1A*, *MTOR* and *NRF1*.** MDA-MB-231 cells were grown in RPMI 1640 media with 1 g/L glucose and treated with 2-deoxy-D-glucose alone or in combination with metformin. After 72 hours, detached cells were reseeded in compound-free media for 72h. Reattached cells were reseeded again and treated with the same compounds. We have performed 4 cycles of detached cell selection. After the last cycle, detached cells were reseeded in compound free media and left to proliferate for 72 hours. RNA expression levels of reattached cells were determined with RNA sequencing. *HIF1A* (A), *MTOR* (B) and *NRF1* (C) gene expressions are shown in more detail. The mean ± SEM for three independent experiments is shown. *p<0.05, **p<0.01, ***p<0.001, ****p<0.0001 versus attached control cells as determined by one-way ANOVA with Dunnett’s post hoc test. FPKM - fragments per kilobase of transcript per million mapped reads. Met – metformin; 2DG - 2-deoxy-D-glucose.

**Figure S21 Analysis of cellular metabolism of actively treated clones**. MDA-MB-231 cells were grown in RPMI 1640 media with 1 g/L glucose and treated with 2-deoxy-D-glucose alone or in combination with metformin or grown on polyHEMA coated plates. After 72 hours, detached cells were reseeded in compound-free media for 72h. Reattached cells were reseeded again and treated with the same compounds. We have performed 4 cycles of detached cell selection. After the last cycle, the attached and detached cells were reseeded in compound free media and the ATP production rates from glycolysis and oxidative phosphorylation (A) were calculated using OCR following injections of oligomycin and rotenone plus antimycin A measured with Seahorse XFe24 analyzer using the Seahorse Real Time ATP Rate Assay. The results were normalized for relative cell number as determined by Hoechst staining. Representative OCR timeline is shown (B). Mean ± SEM is shown for three independent experiments. *p<0.05, **p<0.01, ***p<0.001, ****p<0.0001 versus attached control cells as determined by one-way ANOVA with Dunnett’s post-hoc test. Met – metformin; 2DG - 2-deoxy-D-glucose.

**Figure S22 PCA analysis of genes involved in glycolysis.** MDA-MB-231 cells were grown in RPMI 1640 media with 1 g/L glucose and treated with 2-deoxy-D-glucose alone or in combination with metformin. After 72 hours, detached cells were reseeded in compound-free media for 72h. Reattached cells were reseeded again and treated with the same compounds. We have performed 4 cycles of detached cell selection. After the last cycle, detached cells were reseeded in compound free media and left to proliferate for 72 hours. RNA expression levels of reattached cells were determined with RNA sequencing. PCA analysis of genes involved in glycolysis. The mean for three independent experiments is shown. Met – metformin; 2DG - 2-deoxy-D-glucose.

Figure S23 KEGG pathways analysis of glycolysis. MDA-MB-231 cells were grown in RPMI 1640 media with 1 g/L glucose and treated with 2-deoxy-D-glucose alone or in combination with metformin. After 72 hours, detached cells were reseeded in compound-free media for 72h. Reattached cells were reseeded again and treated with the same compounds. We have performed 4 cycles of detached cell selection. After the last cycle, detached cells were reseeded in compound free media and left to proliferate for 72 hours. RNA expression levels of reattached cells were determined with RNA sequencing. KEGG pathway analysis for glycolysis is shown. The mean for three independent experiments is shown. Met – metformin; 2DG - 2-deoxy-D-glucose.

**Figure S24** **PCA analysis of genes involved in TCA cycle.** MDA-MB-231 cells were grown in RPMI 1640 media with 1 g/L glucose and treated with 2-deoxy-D-glucose alone or in combination with metformin. After 72 hours, detached cells were reseeded in compound-free media for 72h. Reattached cells were reseeded again and treated with the same compounds. We have performed 4 cycles of detached cell selection. After the last cycle, detached cells were reseeded in compound free media and left to proliferate for 72 hours. RNA expression levels of reattached cells were determined with RNA sequencing. PCA analysis of genes involved in TCA cycle. The mean for three independent experiments is shown. Met – metformin; 2DG - 2-deoxy-D-glucose.

Figure S25 KEGG pathway analysis of TCA cycle. MDA-MB-231 cells were grown in RPMI 1640 media with 1 g/L glucose and treated with 2-deoxy-D-glucose alone or in combination with metformin. After 72 hours, detached cells were reseeded in compound-free media for 72h. Reattached cells were reseeded again and treated with the same compounds. We have performed 4 cycles of detached cell selection. After the last cycle, detached cells were reseeded in compound free media and left to proliferate for 72 hours. RNA expression levels of reattached cells were determined with RNA sequencing. KEGG pathway analysis for TCA cycle is shown. The mean for three independent experiments is shown. Met – metformin; 2DG - 2-deoxy-D-glucose.

**Figure S26 Analysis of genes involved in ATP synthase.** MDA-MB-231 cells were grown in RPMI 1640 media with 1 g/L glucose and treated with 2-deoxy-D-glucose alone or in combination with metformin. After 72 hours, detached cells were reseeded in compound-free media for 72h. Reattached cells were reseeded again and treated with the same compounds. We have performed 4 cycles of detached cell selection. After the last cycle, detached cells were reseeded in compound free media and left to proliferate for 72 hours. RNA expression levels of reattached cells were determined with RNA sequencing. Heatmap of expression of genes involved in ATP synthase. Normalized Z score values for three independent experiments are shown in heatmap. Met – metformin; 2DG - 2-deoxy-D-glucose.

**Figure S27** **Transcriptomic analysis of fatty acid metabolism.** MDA-MB-231 cells were grown in RPMI 1640 media with 1 g/L glucose and treated with 2-deoxy-D-glucose alone or in combination with metformin. After 72 hours, detached cells were reseeded in compound-free media for 72h. Reattached cells were reseeded again and treated with the same compounds. We have performed 4 cycles of detached cell selection. After the last cycle, detached cells were reseeded in compound free media and left to proliferate for 72 hours. RNA expression levels of reattached cells were determined with RNA sequencing. Heatmap of expression of genes involved in fatty acid oxidation (A) and fatty acid metabolism (B). Normalized Z score values for three independent experiments are shown in heatmap. *CPT1A* (C), *CPT1B* (D) and *CPT1C* (E) gene expressions are shown in more detail. The mean ± SEM for three independent experiments is shown. *p<0.05, **p<0.01, ***p<0.001, ****p<0.0001 versus attached control cells as determined by one-way ANOVA with Dunnett’s post hoc test. FPKM - fragments per kilobase of transcript per million mapped reads. Met – metformin; 2DG - 2-deoxy-D-glucose.

**Figure S28** **Transcriptomic analysis of genes involved in pyrimidine metabolism.** MDA-MB-231 cells were grown in RPMI 1640 media with 1 g/L glucose and treated with 2-deoxy-D-glucose alone or in combination with metformin. After 72 hours, detached cells were reseeded in compound-free media for 72h. Reattached cells were reseeded again and treated with the same compounds. We have performed 4 cycles of detached cell selection. After the last cycle, detached cells were reseeded in compound free media and left to proliferate for 72 hours. RNA expression levels of reattached cells were determined with RNA sequencing. Heatmap for pyrimidine metabolism (A). PCA analysis of genes involved in pyrimidine metabolism (B). *CAD* (C) and *DHODH* (D) gene expressions are shown in more detail. The mean ± SEM for three independent experiments is shown. Normalized Z score values for three independent experiments are shown in heatmap. *p<0.05, **p<0.01, ***p<0.001, ****p<0.0001 versus attached control cells as determined by one-way ANOVA with Dunnett’s post hoc test. FPKM - fragments per kilobase of transcript per million mapped reads. Met – metformin; 2DG - 2-deoxy-D-glucose.

**Figure S29** **Transcriptomic analysis of genes involved in purine metabolism.** MDA-MB-231 cells were grown in RPMI 1640 media with 1 g/L glucose and treated with 2DG alone or in combination with metformin. After 72 hours, detached cells were reseeded in compound-free media for 72h. Reattached cells were reseeded and treated with the same compounds. We have performed 4 cycles of detached cell selection. After the last cycle, detached cells were reseeded in compound free media and left to proliferate for 72 hours. RNA expression levels of reattached cells were determined with RNA sequencing. Heatmap for purine metabolism (A). PCA analysis of genes involved in purine metabolism (B). *PPAT* (C), *ATIC* (D), *IMPDH1* (E) and *RRM2* (F) gene expressions are shown in more detail. The mean ± SEM for three independent experiments is shown. Normalized Z score values for three independent experiments are shown in heatmap. *p<0.05, **p<0.01, ***p<0.001, ****p<0.0001 versus attached control cells as determined by one-way ANOVA with Dunnett’s post hoc test. FPKM - fragments per kilobase of transcript per million mapped reads. Met – metformin; 2DG - 2-deoxy-D-glucose.

Figure S30 KEGG pathway analysis of purine metabolism. MDA-MB-231 cells were grown in RPMI 1640 media with 1 g/L glucose and treated with 2-deoxy-D-glucose alone or in combination with metformin. After 72 hours, detached cells were reseeded in compound-free media for 72h. Reattached cells were reseeded again and treated with the same compounds. We have performed 4 cycles of detached cell selection. After the last cycle, detached cells were reseeded in compound free media and left to proliferate for 72 hours. RNA expression levels of reattached cells were determined with RNA sequencing. KEGG pathway analysis for purine metabolism is shown. The mean for three independent experiments is shown. Met – metformin; 2DG - 2-deoxy-D-glucose.

**Figure S31** **Transcriptomic analysis of genes involved in pyruvate metabolism.** MDA-MB-231 cells were grown in RPMI 1640 media with 1 g/L glucose and treated with 2-deoxy-D-glucose alone or in combination with metformin. After 72 hours, detached cells were reseeded in compound-free media for 72h. Reattached cells were reseeded again and treated with the same compounds. We have performed 4 cycles of detached cell selection. After the last cycle, detached cells were reseeded in compound free media and left to proliferate for 72 hours. RNA expression levels of reattached cells were determined with RNA sequencing. Heatmap for pyruvate metabolism (A). PCA analysis of genes involved in pyruvate metabolism (B). *PC* (C), *PDHA1* (D) and *PDHB* (E) gene expressions are shown in more detail. The mean ± SEM for three independent experiments is shown. Normalized Z score values for three independent experiments are shown in heatmap. *p<0.05, **p<0.01, ***p<0.001, ****p<0.0001 versus attached control cells as determined by one-way ANOVA with Dunnett’s post hoc test. FPKM - fragments per kilobase of transcript per million mapped reads. Met – metformin; 2DG - 2-deoxy-D-glucose.

Figure S32 KEGG pathway analysis of pyruvate metabolism. MDA-MB-231 cells were grown in RPMI 1640 media with 1 g/L glucose and treated with 2-deoxy-D-glucose alone or in combination with metformin. After 72 hours, detached cells were reseeded in compound-free media for 72h. Reattached cells were reseeded again and treated with the same compounds. We have performed 4 cycles of detached cell selection. After the last cycle, detached cells were reseeded in compound free media and left to proliferate for 72 hours. RNA expression levels of reattached cells were determined with RNA sequencing. KEGG pathway analysis for pyruvate metabolism is shown. The mean for three independent experiments is shown. Met – metformin; 2DG - 2-deoxy-D-glucose.

Figure S33 KEGG pathway analysis of pyruvate metabolism. MDA-MB-231 cells were grown in RPMI 1640 media with 1 g/L glucose and grown on polyHEMA-coated plates. After 72 hours, detached cells were reseeded in compound-free media for 72h. Reattached cells were reseeded again on polyHEMA-coated plates. We have performed 4 cycles of detached cell selection. After the last cycle, detached cells were reseeded in compound free media and left to proliferate for 72 hours. RNA expression levels of reattached cells were determined with RNA sequencing. KEGG pathway analysis for pyruvate metabolism is shown. The mean for three independent experiments is shown. Met – metformin; 2DG - 2-deoxy-D-glucose.

**Figure S34** **Transcriptomic analysis of genes involved in one carbon metabolism.** MDA-MB-231 cells were grown in RPMI 1640 media with 1 g/L glucose and treated with 2-deoxy-D-glucose alone or in combination with metformin. After 72 hours, detached cells were reseeded in compound-free media for 72h. Reattached cells were reseeded again and treated with the same compounds. We have performed 4 cycles of detached cell selection. After the last cycle, detached cells were reseeded in compound free media and left to proliferate for 72 hours. RNA expression levels of reattached cells were determined with RNA sequencing. Heatmap for one carbon metabolism (A). PCA analysis of genes involved in one carbon metabolism (B). *DHFR* (C), *MTHFD1* (D) and *SHMT1* (E) gene expressions are shown in more detail. The mean ± SEM for three independent experiments is shown. Normalized Z score values for three independent experiments are shown in heatmap. *p<0.05, **p<0.01, ***p<0.001, ****p<0.0001 versus attached control cells as determined by one-way ANOVA with Dunnett’s post hoc test. FPKM - fragments per kilobase of transcript per million mapped reads. Met – metformin; 2DG - 2-deoxy-D-glucose.
